# Large Extracellular Vesicle-Derived Latent MMP-8 and Gelatinolytically Active MMP-2 as Potential Circulating Markers for Lymph Node Metastasis in Breast Cancer

**DOI:** 10.3390/cancers18091464

**Published:** 2026-05-02

**Authors:** Liali Yousef Talat, Amr Ahmed WalyEldeen, Ghada Mohamed, Maher H. Ibraheem, Maysaa Mahmoud Maher, Sherif Abdelaziz Ibrahim, Hebatallah Hassan, Martin Götte

**Affiliations:** 1 Department of Zoology, Faculty of Science, Cairo University, Giza 12613, Egypt; liali@sci.cu.edu.eg (L.Y.T.); awalyeldeen@cu.edu.eg (A.A.W.); maysaamaher@cu.edu.eg (M.M.M.); isherif@cu.edu.eg (S.A.I.); 2 Department of Pathology, National Cancer Institute, Cairo University, Cairo 11796, Egypt; dr.ghada.elshafaee@cu.edu.eg; 3 Department of Surgical Oncology, National Cancer Institute, Cairo University, Cairo 11796, Egypt; dr.maherhasan@nci.cu.edu.eg; 4 Department of Surgical Oncology, Baheya Center for Early Detection and Treatment of Breast Cancer, Giza 12511, Egypt; 5 Department of Gynecology and Obstetrics, Münster University Hospital, 48149 Münster, Germany

**Keywords:** breast cancer, lymph node metastasis, extracellular vesicles, matrix metalloproteinases

## Abstract

Background: Lymph node metastasis (LNM) is a determinant of prognosis and in guiding chemotherapy decisions in breast cancer. Herein, we aimed to discover the protease content of the circulating large extracellular vesicles (L-EVs) as potential markers for LNM. Methods: L-EVs were isolated from the plasma of chemotherapy-naïve breast cancer patients with negative LNM (nLNM, n = 40) and positive LNM (pLNM, n = 32) patients using differential centrifugation. The isolated L-EVs were characterized by Transmission Electron Microscopy, dynamic light scattering and EV marker profiling, and their protease content was profiled using unbiased proteome profiler human protease array. Results: Protease profiling uncovered that L-EVs contained significantly elevated levels of MMP-8 (*p* < 0.001) and MMP-9 (*p* < 0.05) in pLNM versus nLNM patients. Further validation by Western blotting confirmed that latent MMP-8 was significantly increased (*p* < 0.01) in L-EVs from pLNM patients. Interestingly, zymography revealed that L-EVs isolated from pLNM patients contained higher levels of latent MMP-9 compared with those from nLNM patients, whereas gelatinolytically active MMP-2 was only detected in L-EVs from pLNM patients and not in those from nLNM. Online datasets revealed that higher *MMP-8* and *MMP-9* mRNA levels were associated with poorer overall, relapse-free, and distant metastasis-free survival. Receiver operating characteristic (ROC) plotter analyses indicated that *MMP-2* and *MMP-8* may serve as predictive biomarkers for response to specific chemotherapeutic regimens. Conclusions: These findings highlight the potential clinical utility of L-EV-derived MMP-2, MMP-8, and MMP-9 expressions and/or activities, as non-invasive blood-based markers associated with nodal progression and therapeutic response.

## Simple Summary

Breast cancer prognosis is strongly influenced by lymph node metastasis (LNM) status. In this study, we examined the protease content of circulating large extracellular vesicles (L-EVs) of breast cancer patients to identify markers associated with LNM. We found that L-EVs from patients with positive LNM (pLNM) contained higher levels of latent matrix metalloproteinase-8 (MMP-8), MMP-9, and active MMP-2 compared with patients with negative LNM (nLNM). Analyses of public cancer databases supported higher MMP-2 and MMP-8 protein expressions in tumor tissues and linked higher *MMP-8* and *MMP-9* mRNA expressions to poorer survival. These findings support further development of L-EV-derived MMP-2, -8, and -9 as blood-based markers associated with nodal progression.

## 1. Introduction

Breast cancer remains a predominant malignancy contributing to cancer-associated mortality in the global female population [1], with lymph node metastasis (LNM) serving as a critical determinant of prognosis and therapeutic decision-making [2]. Accurate differentiation between negative and positive LNM is critical for optimizing clinical decision-making, minimizing unnecessary interventions, and improving patient therapeutic stratification [2,3]. Traditional diagnostic approaches, such as imaging and tissue biopsy, are commonly invasive, time-consuming, and subject to limitations in sensitivity and specificity [4,5]. Over the past decade, liquid biopsy has gained recognition as a minimally invasive and highly promising tool for cancer detection, monitoring, and characterization, leveraging the potential of circulating biomarkers such as extracellular vesicles (EVs) [6].

EVs, secreted by diverse cellular populations, serve as crucial mediators of intercellular signaling and play an active role in promoting tumor progression [7,8]. These vesicles contain a diverse array of functional biomolecules—such as nucleic acids, lipids, and proteins—that serve as indicators of the physiological and pathological states of the cells from which they originate [9,10]. EVs facilitate the formation of pre-metastatic niches by modulating the microenvironment of distant organs, thereby promoting tumor cell colonization [11]. They achieve this by modulating immune responses, altering stromal environments, and facilitating angiogenesis, thereby creating a favorable microenvironment for metastatic seeding [12].

Proteases, a diverse group of enzymes responsible for protein degradation, are fundamentally involved in cancer pathogenesis, with key functions in the processes of invasion and metastasis [9,13]. By facilitating the degradation of the extracellular matrix (ECM), these enzymes enable tumor cells to migrate and spread to distant sites [14,15]. In breast cancer, key proteases such as matrix metalloproteinases (MMPs), cysteine proteases, and serine proteases facilitate tumor development through mechanisms such as enhancing angiogenesis, enabling immune evasion, and conferring resistance to apoptotic signals [14,16,17]. The dysregulation of protease activity has been linked to aggressive tumor phenotypes, poor prognosis, and increased metastatic potential [18,19]. Therefore, uncovering the protease composition within EV cargo provides valuable insights into the metastatic cascade and offers a promising avenue for discovering novel biomarkers of disease progression and therapeutic tailoring.

In this study, we aimed to profile the protease proteome of plasma-derived L-EVs obtained from chemotherapy-naïve breast cancer patients with negative LNM (nLNM) and positive LNM (pLNM) in order to discover potential biomarkers that may facilitate early detection, differentiation of LNM status, guide therapeutic decision-making, and ultimately improve clinical outcomes in breast cancer management.

## 2. Materials and Methods

### 2.1. Clinical Sample Collection

Peripheral blood samples were obtained from 72 female patients who had been diagnosed with primary breast cancer prior to undergoing either breast-conserving surgery or modified radical mastectomy. Eligible participants were adult females (≥18 years), who were histologically confirmed non-metastatic primary breast cancer (stages I–III). None of the included patients had stage IV cancer, autoimmune disorders, or a history of neoadjuvant chemotherapy (were neoadjuvant chemotherapy-naïve). Moreover, any specimens that showed signs of hemolysis or clotting after collection were excluded from the study. The study’s human participant procedures were approved by the Baheya Research Ethics Committee (Giza, Egypt) under Institutional Review Board registration IRB00012829 and were performed in accordance with the Declaration of Helsinki. All participants provided written informed consent. Immediately after surgical excision, breast carcinoma specimens were transferred to DMEM/F12 medium (Gibco, Thermo Fisher Scientific, Waltham, MA, USA) supplemented with 50 μg/mL gentamicin (Lonza, Walkersville, MD, USA) and 1× antibiotic–antimycotic solution (MediaTech Inc., Manassas, VA, USA) on ice [20]. Samples were washed twice with 1× phosphate-buffered saline (PBS) (Lonza, Walkersville, MD, USA) and divided into two portions: one for RNA extraction and the other lyzed in 1× RIPA buffer (50 mM Tris-HCl (Sigma-Aldrich, Steinheim, Germany) pH 7.4, 1% (*v*/*v*) Triton X-100 (Sigma-Aldrich, Steinheim, Germany), 1 mM EDTA (Sigma-Aldrich, Steinheim, Germany), 150 mM NaCl (Sigma-Aldrich, Steinheim, Germany), 0.1% SDS (Sigma-Aldrich, Steinheim, Germany), 1% (*v*/*v*) sodium deoxycholate (Sigma-Aldrich, Steinheim, Germany), supplemented with a protease inhibitor mixture for protein extraction.

### 2.2. Isolation of Large Extracellular Vesicles (L-EVs)

Peripheral blood was drawn into tubes containing acid–citrate–dextrose (ACD) anticoagulant, and platelet-poor plasma (PPP) was obtained by performing two consecutive centrifugation steps at 2500× *g* for 15 min each at room temperature (RT) [21]. EVs were isolated from PPP as previously described [22]. Briefly, PPP samples were processed by high-speed centrifugation at 21,000× *g* for 60 min at 4 °C, enabling efficient sedimentation and recovery of EVs. The resulting pellets were subsequently purified through two consecutive washing steps using prefiltered 0.22 μm PBS by re-centrifugation under the same conditions to minimize residual impurities. In accordance with commonly used EV isolation approaches and the guidelines outlined by the International Society for Extracellular Vesicles (ISEV), the isolated vesicles were operationally defined as L-EVs based on the applied centrifugation conditions, which preferentially enrich vesicles in the larger size range (>200 nm) [23]. The purified pellet fraction was solubilized in 1× RIPA buffer supplemented with a protease inhibitor mixture and sonicated for 20 min. Total protein in isolated L-EVs and cancer tissues was quantified by BCA assay (Serva, Heidelberg, Germany) following the manufacturer’s instructions, with measurements performed on a Nanoquant Tecan Infinite PRO 200 (Tecan, Männedorf, Zürich, Switzerland). For qualitative characterization of the enriched vesicular fraction, a representative L-EV sample and PBS as a negative control were analyzed by flow cytometry. Forward scatter area (FSC-A) and side scatter area (SSC-A) parameters were recorded using an FACS Melody flow cytometer (BD Biosciences, San Jose, CA, USA), and scatter plots were generated using FCS express (version 7). A gate corresponding to the population identified in the L-EV-enriched fraction was defined on the FSC-A versus SSC-A plot, and the same gate was applied to the PBS control [24]. This analysis was used as a qualitative indicator of vesicle enrichment rather than for absolute particle counting. To further assess L-EV enrichment, the protein yield of each sample was normalized to 1 mL of plasma and expressed as µg/mL plasma [25].

### 2.3. Transmission Electron Microscopy (TEM)

Ultrastructural features and size of the isolated L-EVs were analyzed using high-resolution TEM (HR-TEM) at the Electron Microscopy Core Laboratory of the National Research Center (Dokki, Cairo, Egypt). A few microliters of the L-EV suspension prepared in PBS were deposited onto a carbon-coated copper grid and air-dried. Negative staining was performed using 1% phosphotungstic acid, followed by drying at RT. Imaging was then carried out by HR-TEM (JEOL, JEM-2100, Tokyo, Japan) at a 200 kV acceleration potential. The full size of TEM images is provided in Supplementary Materials.

### 2.4. Dynamic Light Scattering (DLS)

The size distribution of the isolated L-EVs and polydispersity index (PDI) were assessed via Zetasizer Nano series instrument (ZEN 3600, Malvern, UK) at the Core Facility of Nanotechnology and Advanced Materials, Agricultural Research Center. For measurement, the L-EV pellet was reconstituted in 3 mL of PBS and vortexed gently to ensure a uniform suspension. Measurements were carried out in triplicate at 25 °C with instrument settings of 378.6 kcps count rate, 4.65 mm measurement position, and a 60 s run time.

### 2.5. Dot Blot and Western Blot Analysis

Dot blot analysis was performed using independently prepared pooled L-EV samples from each experimental group (nLNM and pLNM), with the corresponding post-pelleting supernatants included as negative controls and a cocktail of total human cell lines lysate used as a positive control. Equal amounts of protein in a final volume of 2 µL were directly applied onto nitrocellulose membranes (Amersham, UK), air-dried, and blocked with 5% skimmed milk (Serva, Heidelberg, Germany) prepared in TBST buffer (200 mM Tris, 150 mM NaCl, 0.1% Tween 20). The membranes were then washed thoroughly. Next, primary antibodies against the EV-associated markers CD9, HSP70, and ALIX, as well as the negative marker Calnexin, at a dilution of 1:1000, were applied, and the membranes were incubated at 4 °C overnight. Following the washing steps, appropriate horseradish peroxidase (HRP)-linked secondary antibodies were applied to the membranes for 1 h at RT, and protein visualization was carried out using an enhanced chemiluminescent (ECL) signal (Thermo Scientific, Waltham, MA, USA) and captured with the UVP Biospectrum Imaging System (Analytik Jena, Cambridge, UK).

For Western blot analysis, equivalent amounts of protein from the purified L-EVs and cancer tissues were separated by 10% or 15% SDS-PAGE and then transferred onto a nitrocellulose membrane using a semi-dry electroblotting method [26,27]. For L-EV immunoblotting, samples within each experimental group were pooled into independently prepared pools using equal protein input from individual patients to ensure sufficient protein content for analysis. Accordingly, these experiments were designed to provide exploratory group-level comparisons rather than patient-level assessments of inter-individual variability. In contrast, tissue lysates were analyzed individually, with 12 samples from each group (nLNM and pLNM). Western blot was carried out under the same blocking, antibody incubation, and detection conditions described for the dot blot procedure, using primary antibodies against MMP-8, CD9, and β-actin at a dilution of 1:1000. CD9 was utilized as the reference marker to normalize protein loading for L-EVs in the Western blot analysis, with the blots prepared simultaneously under non-reducing conditions, and β-actin was utilized for tissue samples. The antibodies used in this study are listed in Supplementary Table S1, and the original full-length uncropped nitrocellulose membranes are provided in Supplementary Materials.

### 2.6. Proteome Profiler Human Protease Array

L-EV protease profiling was conducted utilizing the Proteome Profiler Human Protease Array Kit (ARY021B, R&D Systems, Abingdon, UK). For each experimental group (nLNM and pLNM), L-EV samples were pooled into independently prepared pooled samples by combining equal amounts of protein derived from individual patients to ensure sufficient protein content for analysis. A total of 100 µg of protein, as quantified by a BCA assay, was diluted in 1.5 mL Array Buffer 6 and 15 µL of protease detection antibody cocktail. Array membranes were pre-blocked for 1 h at RT with 2 mL of Array Buffer 6 to minimize non-specific binding, then incubated overnight at 4 °C with the prepared L-EV samples under continuous shaking. After overnight incubation, the membranes were subjected to three consecutive washes for 10 min with 1× washing buffer and were subsequently incubated 30 min with 1× streptavidin–HRP at RT. Following additional washing steps, the signal was developed using a Chemi Reagent Mix and detected with the UVP Biospectrum Imaging System. The intensity of each duplicated spot was measured using ImageJ software (version 1.53c) and normalized to the positive control spots on each membrane. The fold change was calculated as the ratio of the mean normalized spot intensity in the pLNM group to that in the nLNM group. Given that the analysis was performed on independently pooled samples, these comparisons should be considered exploratory rather than patient-level statistical inferences. Original protease array images are provided in Supplementary Materials.

### 2.7. Zymography Assay

Gelatin zymography was performed to evaluate gelatinolytic activity in whole plasma, purified L-EV fractions and their corresponding post-pelleting supernatants. For plasma, representative individual samples from the nLNM and pLNM groups were analyzed, whereas for the L-EV fraction and supernatant fractions, independently prepared pooled samples were analyzed. Equal protein concentrations (with 20 µg) were separated on a 7.5% or 10% SDS-PAGE containing 0.1% gelatin under cold conditions. The gels were subsequently rinsed twice at RT for 15 min per wash using 2.5% Triton X-100 to eliminate residual detergent. Gels were incubated overnight at 37 °C in buffer (50 mM Tris, pH 7.8; 5 mM CaCl_2_ (Sigma-Aldrich, Steinheim, Germany); 0.05% Brij 35 (Sigma-Aldrich, Steinheim, Germany) and then stained with Coomassie Brilliant Blue, de-stained to reveal zones of proteolytic activity, and imaged using a UVP BioSpectrum Imaging System. Original zymography images are provided in Supplementary Materials.

### 2.8. RNA Isolation and Quantitative Real-Time PCR (qRT-PCR) Analysis

Freshly excised tumor tissues were processed for total RNA extraction using QIAzol lysis reagent (Qiagen, Hilden, Germany) and the GeneJET RNA Purification Kit (Thermo Fisher Scientific, Vilnius, Lithuania) following the manufacturer’s guide. The concentration and quality of extracted RNA were evaluated with the NanoQuant Infinite^®^200 PRO system (Tecan, Männedorf, Zürich, Switzerland). For cDNA synthesis, 1 µg of total RNA was reverse-transcribed with the cDNA Reverse Transcription Kit (Thermo Fisher Scientific, Vilnius, Lithuania). Subsequently, the mRNA expression of *MMP-2* and *MMP-8* was assessed in tumor specimens from nLNM (n = 9) and pLNM (n = 13) by SYBR™ Green-based qPCR (SYBR™ Green PCR Master Mix; Thermo Fisher Scientific, Vilnius, Lithuania) performed on the StepOnePlus Real-Time PCR System (Applied Biosystems, Foster City, CA, USA). Relative transcript levels were determined using the 2^−ΔΔCt^ method, as described previously [28]. Gene expression was normalized against 18S rRNA (housekeeping reference), and primer sequences are reported in Supplementary Table S2.

### 2.9. In Silico Exploration Using Available Biological Data Sources

Vesiclepedia (http://www.microvesicles.org) (accessed on 1 January 2026) was queried to verify prior reports of EV-associated proteases identified in the protease array and to retrieve the associated supporting studies [29]. Transcriptional expression levels of *MMP-2* and *MMP-8* across different LNM stages (N0–N3) in breast carcinoma tissues were analyzed using The Cancer Genome Atlas (TCGA) data available through the UALCAN database (http://ualcan.path.uab.edu/) (accessed on 1 January 2026) [30]. The Human Protein Atlas immunohistochemistry (IHC) datasets were further analyzed to assess MMP-2, MMP-8, and MMP-9 protein expression and localization in normal breast tissue and breast tumors (HPA; https://www.proteinatlas.org/) (accessed on 1 January 2026) [31,32]. The prognostic significance of *MMP-2* (201069_at), *MMP-8* (231688_at), and *MMP-9* (203936_at) mRNA expression in breast cancer was explored using the Kaplan–Meier Plotter (https://www.kmplot.com/analysis/) (accessed on 1 January 2026), which integrates comprehensive patient datasets [33]. The analysis covered the breast cancer cohort divided based on lymph node (LN) status. Survival outcomes—including overall survival (OS), relapse-free survival (RFS), and distant metastasis-free survival (DMFS)—were evaluated together with hazard ratios (HRs), confidence intervals (CIs), and log-rank *p*-values, as obtained from the database for comprehensive statistical analysis. Transcriptomic profiles of chemotherapy-responsive and non-responsive breast cancer patients were collected and analyzed using receiving operating characteristics (ROC) Plotter (https://rocplot.com/site/treatment) (accessed on 1 January 2026) to assess the predictive relevance of *MMP-8* (231688_at), *MMP-2* (201069_at), and *MMP-9* (203936_at) expression for chemotherapy response in terms of pathological complete response (pCR). The platform reports the area under the curve (AUC) value, *p*-value and an optimal expression cutoff to distinguish responders from non-responders [34].

### 2.10. Statistical Analysis

Statistical analyses were performed using SPSS (version 27). Data normality was initially assessed by evaluating skewness and kurtosis. For normally distributed data, mean differences between two independent groups were evaluated using an independent sample Student’s *t*-test. In cases where non-parametric analysis was needed, the Chi-square test was applied to evaluate clinicopathological variables. Additionally, differences in mean values across more than two groups were evaluated using one-way analysis of variance (ANOVA). Results are presented as mean ± SEM, with statistical significance set at *p* < 0.05.

## 3. Results

### 3.1. Clinicopathological Characteristics of Breast Cancer Patients Grouped by LNM

The study population of 72 breast cancer patients who had not received prior chemotherapy was stratified according to LNM status into two cohorts: nLNM (n = 40) and pLNM (n = 32). Table 1 presents the clinicopathological features of the two groups. Statistical analysis revealed no significant differences in age, menopausal status, laterality, tumor size, histological grade, hormone receptor expression (ER and PR), HER2 status, and tumor classification (T stage) (*p* > 0.05 for all parameters).

### 3.2. Characterization of Isolated Plasma-Derived L-EVs

**Figure 1:**
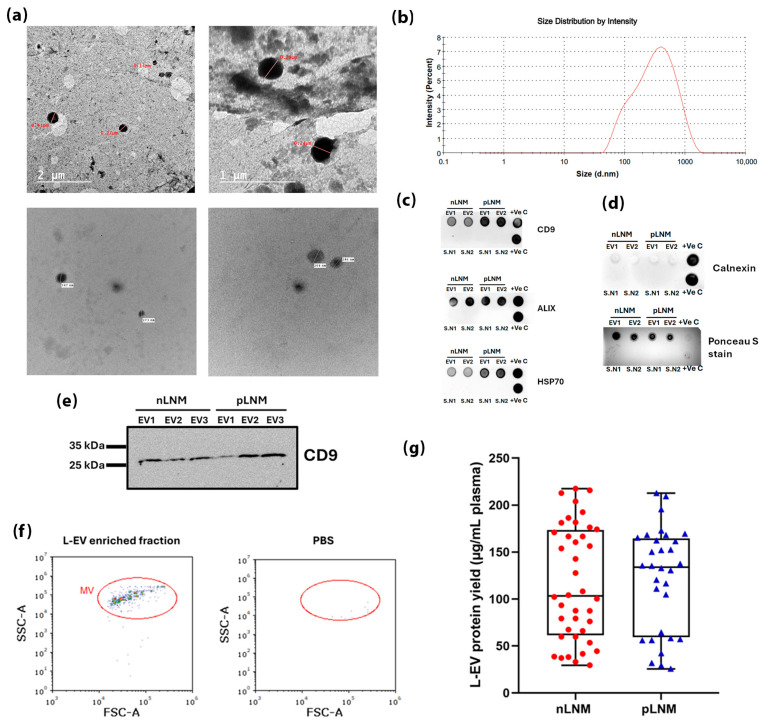
Characterization of plasma-derived large extracellular vesicles (L-EVs). (**a**) High-resolution Transmission Electron Microscopy (HR-TEM) images of representative independent samples showing intact L-EVs with an average size ˃ 200 nm. (**b**) Size distribution of representative independent L-EV sample by dynamic light scattering (DLS). (**c**) Dot blot of EV markers CD9, HSP70, ALIX, and (**d**) non-EV marker Calnexin in L-EV samples using independently pooled samples from each group (nLNM: EV1 (n = 10) and EV2 (n = 10); pLNM: EV1 (n = 8) and EV2 (n = 8)), corresponding post-pelleting supernatants (S.N1 and S.N2) and a cocktail of total human cell lines lysate (+Ve C); Ponceau S stain of the Calnexin membrane serving as a total protein loading. (**e**) Western blot of CD9 in independently pooled L-EV samples from each group (nLNM: EV1 (n = 6), EV2 (n = 7) and EV3 (n = 7); pLNM: EV1 (n = 5), EV2 (n = 5) and EV3 (n = 6)). Original full-length uncropped nitrocellulose membranes of biological duplicates are presented in Supplementary Materials, Figures S1–S15. (**f**) Representative flow cytometric forward scatter area (FSC-A) versus side scatter area (SSC-A) plots of an L-EV-enriched fraction and PBS as a negative control. (**g**) L-EV protein yield normalized to 1 mL of plasma in the nLNM (n = 40) and pLNM (n = 32) groups, expressed as µg/mL plasma.

The successful isolation of plasma-derived L-EVs from breast cancer patients was confirmed by multiple structural and molecular analytical techniques. HR-TEM was used to assess the morphology and size of the isolated L-EVs. As shown in Figure 1a, the vesicles appeared as cup-shaped, intact structures with an average size ˃ 200 nm, confirming their structural integrity. DLS analysis further showed a predominant vesicle population within the L-EV size range, with a major peak at 390.3 nm and a PDI of 0.390, indicating a relatively homogeneous size distribution (Figure 1b). The isolated vesicles were defined as L-EVs in accordance with the applied centrifugation protocol and in line with ISEV recommendations, a classification that was further supported by the HR-TEM and DLS results.

EV marker profiling by dot blot confirmed enrichment of CD9, HSP70 and ALIX in the isolated L-EV fraction (Figure 1c), while the negative EV marker Calnexin was undetectable (Figure 1d). In addition, the absence of EV marker signals in the corresponding post-pelleting supernatants further supports the successful isolation of the vesicular fraction. Further validation by Western blot analysis revealed the existence of CD9 with the expected molecular weight (~25 kDa) in the L-EV fraction (Figure 1e). Collectively, these results confirm the successful and efficient isolation of plasma-derived L-EVs suitable for downstream analysis.

Representative flow cytometric analysis was performed as a qualitative characterization approach and revealed a distinct vesicle population in the L-EV-enriched fraction that was clearly distinguishable from the minimal background signal observed in the PBS negative control (Figure 1f). Quantification of protein content in plasma-derived L-EV samples showed comparable normalized protein yield between the nLNM and pLNM groups, with mean values of 116.8 ± 9.587 and 120.1 ± 9.811 µg/mL plasma, respectively, indicating no substantial difference in overall L-EV enrichment (Figure 1g).

### 3.3. Protease Profiling Reveals a Significant Elevation of MMP-8 and MMP-9 in L-EVs Breast Cancer Patients with pLNM Compared with nLNM

**Figure 2:**
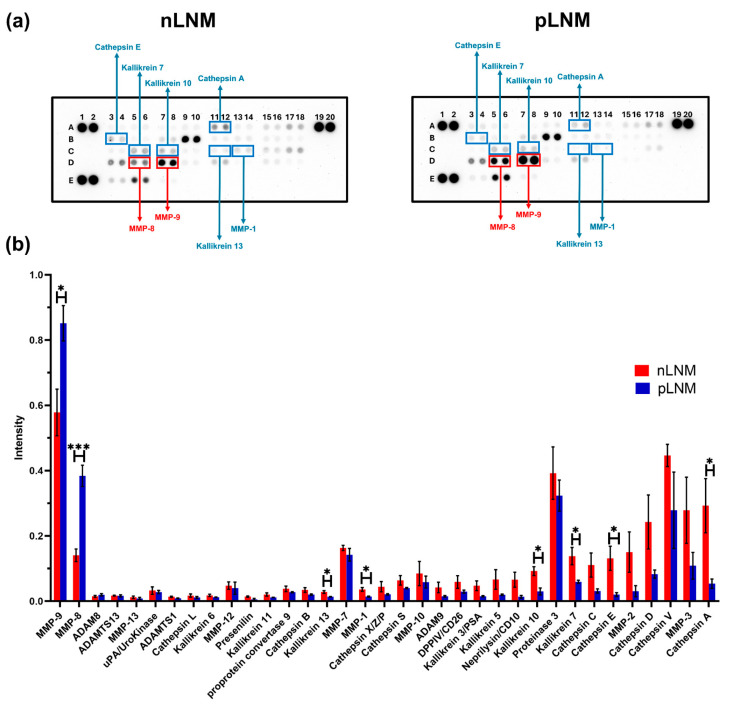
Protease profiling of plasma-derived large extracellular vesicles (L-EVs) from breast cancer patients. (**a**) Representative protease array membranes showing expression patterns in patients with nLNM and pLNM groups using screening cohort of two independently prepared pooled L-EV samples from each group (nLNM: n = 5 each; pLNM: n = 5 each). Blue boxes indicate decreased proteases, while red boxes indicate elevated proteases. (**b**) Quantitative analysis of protease signal intensity in nLNM and pLNM groups. Original protease array images of biological duplicates are presented in Supplementary Materials, Figures S16 and S17. Bars represent means ± SEM. * *p* < 0.05; *** *p* < 0.001. *p*-values were determined using Student’s *t*-test.

To discover the protease cargo of plasma-derived L-EVs in relation to LNM status, we employed the proteome profiler human protease array. This platform enabled the simultaneous detection of 35 proteases within EV cargo isolated from patients with nLNM and pLNM. Notably, the array-based analysis showed an overall reduction in the levels of most proteases in L-EVs derived from pLNM patients compared with nLNM controls. (Figure 2a,b). Notably, MMP-8 (*p* < 0.001) and MMP-9 (*p* < 0.05) were the only proteases significantly upregulated in L-EVs from pLNM patients relative to nLNM patients, each showing a fold change ≥ 1.5. In contrast, the levels of cathepsin A (*p* < 0.05), cathepsin E (*p* < 0.05), kallikrein-7 (*p* < 0.05), kallikrein-10 (*p* < 0.05), kallikrein-13 (*p* < 0.05), and MMP-1 (*p* < 0.05) were significantly downregulated in L-EVs from pLNM patients compared with those from nLNM patients, each showing a fold change ≤ 0.5 (Table 2).

### 3.4. Differential Expression of Latent and Active MMP-8 in L-EVs from pLNM Compared with nLNM Breast Cancer Patients

**Figure 3:**
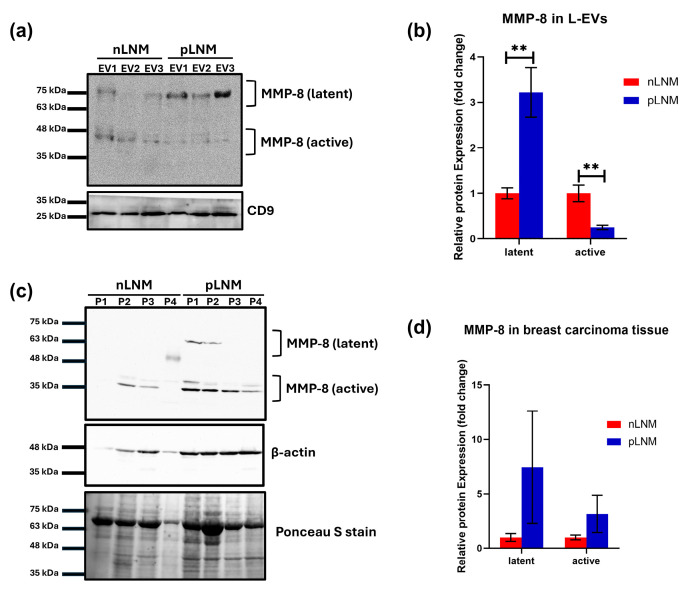
MMP-8 expression in large extracellular vesicles (L-EVs) and tumor tissues in breast cancer patients with nLNM and pLNM. (**a**) Western blot analysis of latent and active MMP-8 in L-EVs derived from nLNM and pLNM patients using independently prepared pooled samples from each group (nLNM: EV1 (n = 4), EV2 (n = 5) and EV3 (n = 5); pLNM: EV1 (n = 3), EV2 (n = 3) and EV3 (n = 4)); CD9 serving as a loading control. (**b**) Quantitative analysis of MMP-8 band intensities in L-EVs. (**c**) Western blot of MMP-8 in tumor tissues of breast cancer patients with nLNM (n = 4) and pLNM (n = 4); β-actin serving as a loading control; Ponceau S staining of the corresponding membrane as a total protein loading. P1-P4: patient samples 1-4. (**d**) Quantitative analysis of MMP-8 band intensities in tumor tissue. Original full-length uncropped nitrocellulose membranes of biological replicates are presented in Supplementary Materials, Figures S18–S30. Bars represent means ± SEM. ** *p* < 0.01. *p*-values were determined using Student’s *t*-test.

Given the observed elevation of MMP-8 in L-EVs from breast cancer patients with pLNM, we next verified its expression by Western blot. A pronounced increase in the latent form of MMP-8 was observed in L-EVs from patients with pLNM relative to those with nLNM, whereas the active form of MMP-8 in L-EVs was significantly reduced in the pLNM group (both *p* < 0.01) (Figure 3a,b). In tumor tissue samples, no consistent differences were detected in the levels of latent and active MMP-8 between patients with nLNM and pLNM (Figure 3c,d).

### 3.5. MMP-2 and MMP-9 Activities Are Elevated in L-EVs from pLNM Compared with nLNM Breast Cancer Patients

**Figure 4:**
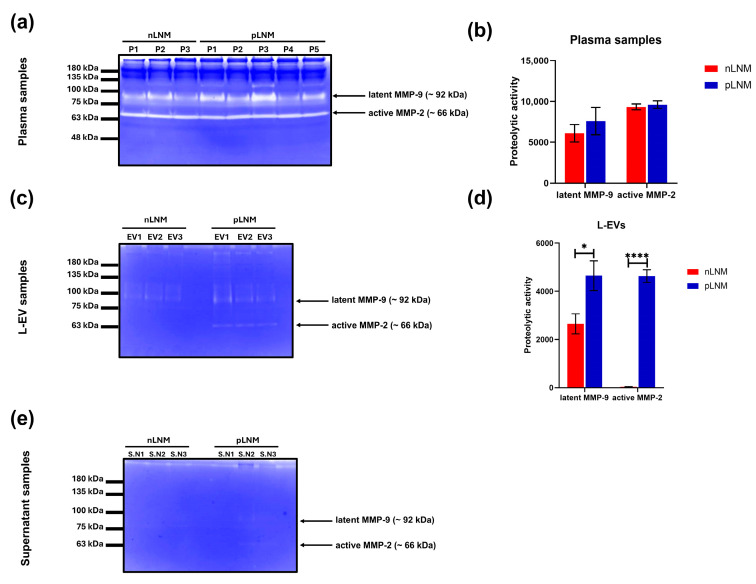
Gelatin zymography analysis of MMP-2 and MMP-9 enzymatic activity in plasma, large extracellular vesicles (L-EVs), and their corresponding supernatant samples from nLNM and pLNM groups. (**a**) Representative gelatin zymography images showing the gelatinolytic activity of latent MMP-9 (~92 kDa) and active MMP-2 (~66 kDa) in plasma samples derived from nLNM (n = 3) and pLNM (n = 5) patients. P1-P4: patient samples 1-4. (**b**) Quantitative analysis of latent MMP-9 and active MMP-2 activities in plasma samples. (**c**) Representative gelatin zymography images showing the gelatinolytic activity of latent MMP-9 (~92 kDa) and active MMP-2 (~66 kDa) in L-EV samples derived from nLNM and pLNM patients using independently prepared pooled samples from each group (nLNM: EV1 (n = 6), EV2 (n = 7) and EV3 (n = 7); pLNM: EV1 (n = 5), EV2 (n = 5) and EV3 (n = 6)). (**d**) Quantitative analysis of latent MMP-9 and active MMP-2 activities in L-EVs. (**e**) Gelatin zymography of supernatant fractions showing no detectable gelatinolytic activity for both enzymes. Original zymography images of biological duplicates are presented in Supplementary Materials, Figures S31–S33. Bars represent means ± SEM. * *p* < 0.05; **** *p* < 0.0001. *p*-values were determined using Student’s *t*-test.

Considering the significant elevation of MMP-9 content revealed by the protease array in L-EVs isolated from pLNM patients relative to nLNM patients, we next sought to examine whether these changes translated into functional differences in enzymatic activity. Gelatin zymography was performed for whole plasma, L-EV fractions, and their corresponding post-pelleting supernatants. In plasma samples, distinct gelatinolytic bands corresponding to latent MMP-9 (~92 kDa) and active MMP-2 (~66 kDa) were observed in both the nLNM and pLNM groups, with no statistically significant differences in the activity levels of both MMP-2 and MMP-9 (Figure 4a,b). In contrast, L-EV samples exhibited marked differences between the nLNM and pLNM groups. The latent form of MMP-9 was detected in both L-EVs from nLNM and pLNM (Figure 4c), with significantly higher activity in patients with pLNM (*p* < 0.05). Interestingly, active MMP-2 was exclusively identified in L-EVs from patients with pLNM, whereas its activity was not detected in those with nLNM (Figure 4d; *p* < 0.0001). To rule out the possibility that the soluble plasma fraction may contain MMP traces that affect the zymographic results, we have performed zymography for corresponding post-pelleting supernatants of the L-EVs. Notably, no gelatinase activities for either MMP-2 or MMP-9 were detected (Figure 4e), indicating that the majority of these proteases are confined within the L-EV fractions rather than the supernatant fractions.

### 3.6. Expression of MMP-2, MMP-8, and MMP-9 in Breast Cancer and Its Relation with LNM Status

**Figure 5:**
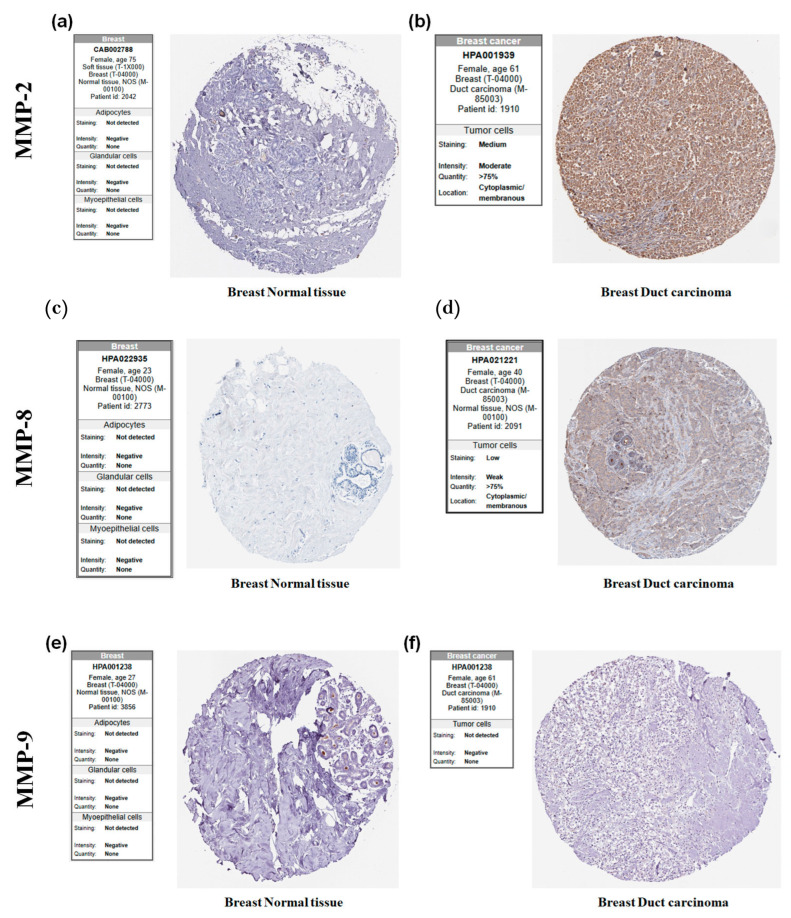
Immunohistochemical (IHC) analysis of MMP-2, MMP-8 and MMP-9 expressions in breast tissues from the Human Protein Atlas database (HPA; https://www.proteinatlas.org/) (accessed on 1 January 2026). (**a**) Representative IHC images showing the MMP-2 expression in normal breast tissue (**b**) and in ductal carcinoma tissue. (**c**) Representative IHC images showing the MMP-8 expression in normal breast tissue (**d**) and in breast ductal carcinoma tissue. (**e**) Representative IHC images showing the MMP-9 expression in normal breast tissue (**f**) and in breast ductal carcinoma tissue.

To evaluate differences in MMP-2, MMP-8, and MMP-9 protein expression between normal breast tissues and ductal carcinoma, we analyzed corresponding IHC datasets available through the HPA database. As shown in Figure 5a, MMP-2 staining was undetectable in normal breast tissues, with no immunoreactivity observed in either glandular or myoepithelial cells. However, in breast ductal carcinoma, MMP-2 showed moderate cytoplasmic and membranous positivity in more than 75% of tumor cells (Figure 5b). Similarly, MMP-8 staining was not detected in normal breast tissue (Figure 5c), whereas breast ductal carcinoma tissue exhibited weak yet detectable expressions of MMP-8, with over 75% of tumor cells showing positive cytoplasmic and membranous localization (Figure 5d). Conversely, MMP-9 showed no detectable staining in either normal breast tissue (Figure 5e) or breast ductal carcinoma tissue (Figure 5f).

**Figure 6:**
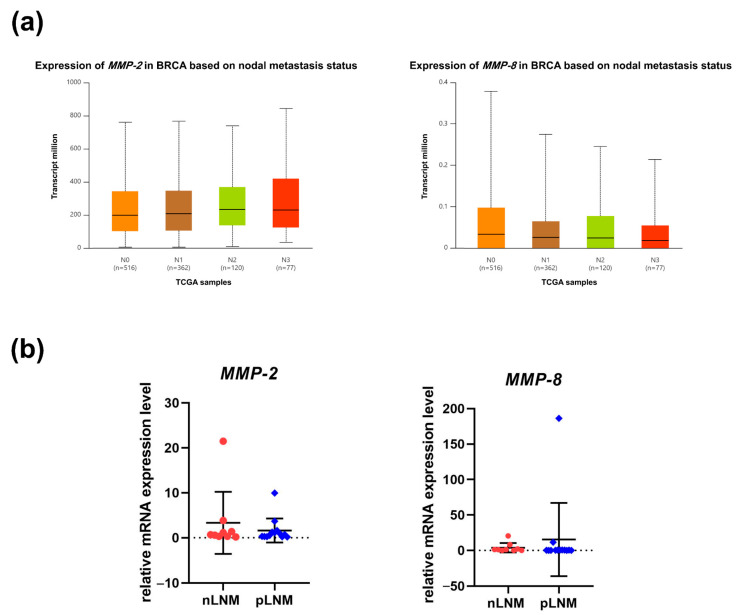
*MMP-2* and *MMP-8* mRNA expression in breast cancer according to nodal metastasis. (**a**) UALCAN-based analysis showing *MMP-2* (**left**) and *MMP-8* (**right**) transcript levels in breast carcinoma tissues stratified by nodal stage (N0–N3) (http://ualcan.path.uab.edu/) (accessed on 1 January 2026). (**b**) qPCR validation of *MMP-2* (**left**) and *MMP-8* (**right**) mRNA levels in breast tumor tissues, comparing patients with pLNM (n = 13) to those with nLNM (n = 9).

Next, expression data retrieved from TCGA-derived were accessed through UALCAN to evaluate *MMP-2* and *MMP-8* transcript abundance in breast cancer tissues according to nodal metastatic status. We then confirmed the tissue-level expression of *MMP-2* and *MMP-8* by qPCR in surgically resected tumors from nLNM and pLNM cohorts. Consistent with TCGA transcriptomic data analysis (Figure 6a), in tumor tissue specimens, *MMP-2* and *MMP-8* mRNA expression did not differ significantly between the nLNM and pLNM groups (Figure 6b).

### 3.7. Prognostic Significance of MMP-2, MMP-8, and MMP-9 Expression in Breast Cancer Patients

**Figure 7:**
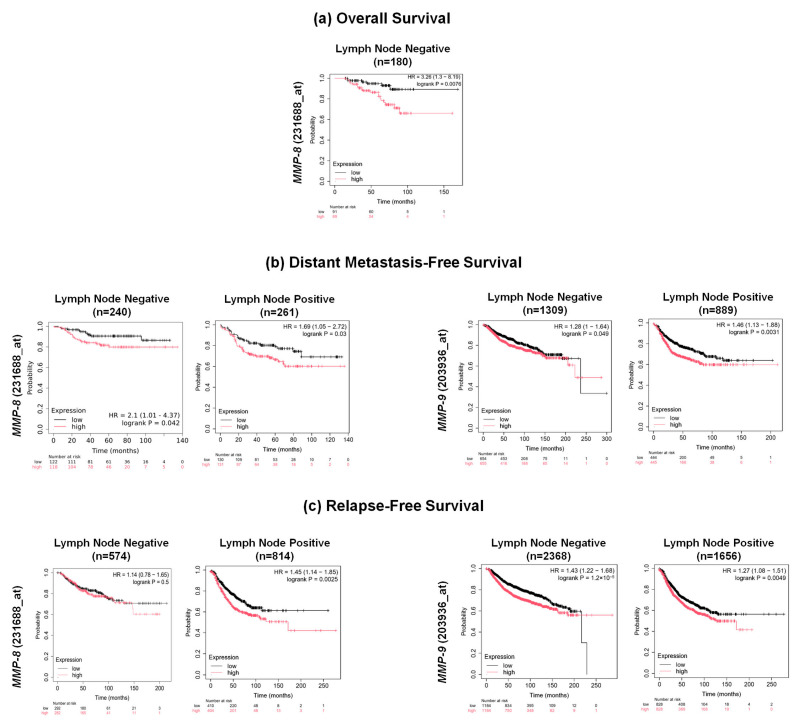
Prognostic significance of *MMP-8*, and *MMP-9* mRNA expression in breast cancer patients using the Kaplan–Meier Plotter (https://www.kmplot.com/analysis/) (accessed on 1 January 2026). Kaplan–Meier survival curves showing overall survival (**a**), distant metastasis-free survival (**b**), and relapse-free survival (**c**) in breast cancer patients sub-grouped by lymph node (LN) status.

Kaplan–Meier survival analyses were performed using publicly available datasets to determine whether mRNA expression of *MMP-2* (201069_at), *MMP-8* (231688_at), and *MMP-9* (203936_at) was associated with OS, DMFS, and RFS in breast cancer patients stratified by LN status (Figure 7).

In patients with nLNM, elevated *MMP-8* expression was significantly correlated with poorer OS (*p* = 0.0076, n = 180), whereas *MMP-2* and *MMP-9* levels showed no significant association with OS (Figure 7a). Elevated transcript levels of both *MMP-8* and *MMP-9* was found to be significantly associated with decreased DMFS in nLNM patients (*MMP-8*: *p* = 0.042, n = 240; *MMP-9*: *p* = 0.049, n = 1309) and in pLNM patients (*MMP-8*: *p* = 0.03, n = 261; *MMP-9*: *p* = 0.0031, n = 889). No significant correlation was observed between *MMP-2* expression and DMFS (Figure 7b). Furthermore, increased expression of *MMP-8* and *MMP-9* was associated with poorer RFS in nLNM patients (*MMP-8*: *p* = 0.5, n = 574; *MMP-9*: *p* < 0.0001, n = 2368) and in pLNM patients (*MMP-8*: *p* = 0.0025, n = 814; *MMP-9*: *p* = 0.0049, n = 1656), whereas *MMP-2* expression showed no significant association with RFS (Figure 7c).

### 3.8. MMP-2 and MMP-8 as Predictive Biomarkers for Chemotherapy Response in Breast Cancer Patients

**Figure 8:**
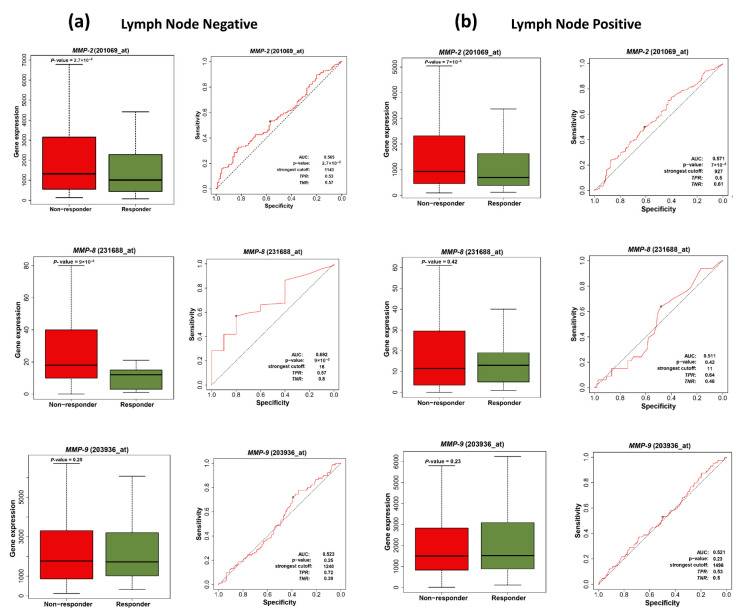
ROC Plotter-based evaluation of *MMP-2*, *MMP-8*, and *MMP-9* expression for predicting chemotherapy responsiveness in breast cancer patients (https://rocplot.com/site/treatment; accessed on 1 January 2026). Associations between *MMP-2*/*MMP-8*/*MMP-9* mRNA abundance and therapeutic response are presented as boxplots with corresponding ROC curves for lymph node-negative (**a**) and lymph node-positive (**b**) groups. The figure includes AUC values, statistical significance (*p*-values), the best-performing threshold, and diagnostic indices (TPR and TNR).

To explore the predictive potential of *MMP-2* (201069_at), *MMP-8* (231688_at), and *MMP-9* (203936_at), expressions in response to chemotherapy, transcriptomic data from breast cancer patients stratified by LN status were analyzed using the ROC Plotter platform (Figure 8).

In nLNM patients, a statistically significant elevation in *MMP-2* and *MMP-8* expressions was observed in non-responders compared with responders (*MMP-2*: *p* = 0.027; *MMP-8*: *p* = 0.009), whereas no significant variation in *MMP-9* expression was observed between the two cohorts (*p* = 0.25). In addition, ROC curve analysis further supported the predictive relevance of *MMP-2* and *MMP-8* expressions, with an AUC of 0.565 and 0.692, respectively, suggesting that *MMP-2* and *MMP-8* exhibit predictive potential in chemotherapy response (Figure 8a). In contrast, within the pLNM group, only *MMP-2* expression showed a statistically significant elevation in non-responders compared with responders (*p* = 0.007), whereas *MMP-8* and *MMP-9* displayed no significant expression differences between the two groups (*MMP-8*: *p* = 0.42; *MMP-9*: *p* = 0.23). Consistently, ROC curve analysis reflected the predictive ability of *MMP-2*, with an AUC of 0.571 (Figure 8b).

Further assessment of *MMP-2*, *MMP-8* and *MMP-9* expression profiles in breast cancer patients with nLNM and pLNM, subjected to different chemotherapy regimens such as FEC (fluorouracil, epirubicin, and cyclophosphamide), anthracycline-based therapy, FAC (fluorouracil, adriamycin, and cyclophosphamide), and taxane was performed to determine their predictive associations with treatment response (Table 3). In the nLNM group, higher expression of *MMP-2* was associated with resistance to FEC treatment (*p* = 0.0043, AUC = 0.665), whereas no significant association was observed with other regimens. Similarly, increased *MMP-8* expression correlated with reduced chemotherapy response in anthracycline treatment (*p* = 0.009, AUC = 0.692), but not in other treatment subtypes. For *MMP-9*, none of the chemotherapy regimens in nLNM patients demonstrated statistical significance.

In the pLNM cohort, *MMP-2* continued to exhibit predictive significance, particularly among patients treated with anthracycline or taxane regimens (*p* = 0.0073, AUC = 0.57; *p* = 0.0026, AUC = 0.586, respectively), whereas *MMP-8* showed no significant association with treatment response in pLNM cases. Notably, higher *MMP-9* expression was linked to better response to FEC therapy (*p* = 0.003, AUC = 0.629), whereas no significant associations were detected for the remaining treatment regimens.

## 4. Discussion

This study reveals that plasma-derived L-EVs from chemotherapy-naïve breast cancer patients exhibit distinct protease expression profiles associated with LNM. Notably, MMP-8 showed a differential expression pattern, with its latent form increased in L-EVs across advancing nodal stage, whereas the active form showed the opposite trend, suggesting an inverse regulation and potential redistribution of its proteolytic activity during nodal progression in breast cancer. Additionally, MMP-2 and MMP-9 exhibited elevated enzymatic activities within L-EVs isolated from patients with pLNM compared with nLNM, highlighting their involvement in metastatic progression.

EV cargo has been shown to contain a diverse array of protease enzymes, including several MMP subtypes such as soluble MMPs, membrane-associated MMPs, and members of the ADAM and ADAMTS proteolytic families. These enzymes can mediate ECM remodeling, thereby influencing tissue architecture, regeneration, and disease progression, including cancer, and consequently represent potential therapeutic targets [35,36]. Interestingly, our finding of reduced protease content within circulating L-EVs of pLNM patients compared with nLNM patients may be explained by the preferential accumulation of tumor-derived EVs in lymph nodes, as previously reported in melanoma models, where EVs were found to accumulate in tumor-draining lymph nodes rather than in circulation, contributing to lymphatic niche remodeling [37]. This may suggest a similar EV-driven mechanism in breast cancer, with protease-rich vesicles retained in lymph nodes for microenvironmental conditioning and metastatic dissemination. Among the proteases analyzed, only MMP-8 and MMP-9 exhibited significant elevations in pLNM compared with nLNM patients. Together, these patterns draw attention to the distinctive regulatory profiles of MMP-8 and MMP-9, positioning them as key molecules in subsequent analyses of nodal advancement.

MMP-8, commonly referred to as collagenase-2, is predominantly synthesized by neutrophils and has been implicated in a range of inflammation-related pathologies [38]. Dysregulated MMP-8 has been reported to contribute to the progression and unfavorable clinical outcomes across multiple malignancies, including head and neck [39], ovarian [40], colorectal [41], and hepatocellular carcinomas [42]. This consistent association of MMP-8 expression with tumor progression strongly supports its potential utility as a biomarker for prognosis and a key mediator of cancer invasiveness. In breast cancer, MMP-8 expression was found to be elevated in both serum and tumor tissues compared with healthy controls [43,44], a pattern that aligns with the IHC results from the HPA database showing complete absence of MMP-8 staining in normal breast tissue but weak, widespread cytoplasmic and membranous expression in breast ductal carcinoma cells. Nevertheless, our Western blot analysis showed no significant difference for the levels of latent and active forms of MMP-8 in breast carcinoma tissues of pLNM and nLNM patients, whereas a significant increase in the latent form of MMP-8 was detected in plasma-derived L-EVs from pLNM compared with nLNM patients. This discrepancy suggests that EV-associated MMP-8 may not directly reflect overall tissue expression but rather selective sorting and release from biologically active cellular subsets within the tumor microenvironment (TME), including stromal and inflammatory compartments, that contribute to the circulating EV pool [45] and may not be captured by bulk tissue homogenates [46]. A similar discordance has been reported in lung cancer, where blood exosome PD-L1 was significantly associated with LNM, whereas tumor tissue PD-L1 assessed by IHC showed no significant association with clinicopathological features [47].

Unlike cell-surface MMPs, which primarily mediate local and autocrine ECM remodeling, EV-associated MMPs function mainly at distant sites, where they influence stromal and microenvironmental cells and contribute to ECM degradation upon delivery to the TME [48,49]. Moreover, MMP-8 is secreted as an inactive pro-enzyme whose activation is tightly regulated and primarily occurs at sites of inflammation [50]. Taken together, these observations support the hypothesis that L-EVs may serve as vehicles for transporting latent MMP-8 to metastatic or inflammatory sites, where it can be locally activated and contribute to ECM remodeling, thereby promoting cancer progression.

Apart from MMP-8, the protease array results also indicated increased MMP-9 in L-EVs from pLNM patients, prompting further assessment of its enzymatic activity using gelatin zymography. The results revealed enrichment of latent MMP-9 and a pronounced elevation of active MMP-2 within L-EVs from pLNM patients compared with nLNM patients. MMP-2 and MMP-9, key members of the gelatinase subgroup, break down type IV/V collagens in the ECM and basement membrane [51], and their overexpression has been correlated with increased tumor aggressiveness, advanced disease stage, LNM, and reduced survival in breast cancer patients [52]. Importantly, previous studies have reported that elevated MMP-2 and MMP-9 expressions are closely linked to enhanced lymphatic invasion and LNM across multiple cancer types, as their experimental inhibition was shown to suppress angiogenesis and lymphangiogenesis, thereby reducing nodal metastasis [49]. The presence of these gelatinases in L-EVs may indicate an EV-driven mechanism that promotes ECM degradation and lymphatic remodeling, facilitating nodal metastasis. Similar EV-driven enrichment of MMP-2 and MMP-9 has been described in breast cancer [53] as well as in prostate [54], fibrosarcoma [55], and ovarian carcinoma cells [56], where these vesicular proteases facilitate tumor cell migration and metastatic spread. A previous study on colorectal cancer reported distinct activation profiles of MMP-2 and MMP-9, where active MMP-2 was markedly increased in tumor tissues, whereas MMP-9 accumulated predominantly in its latent proform [57]. This difference has been attributed to the fact that MMP-2 activation mainly occurs within tumor cells through membrane-type activators such as MT1-MMP, while MMP-9 is largely secreted by infiltrating immune cells, including macrophages and neutrophils, in its pro-enzymatic form. Supporting this concept, elevated levels and enzymatic activity of latent MMP-9 have been documented in highly aggressive ovarian tumors, where its presence is thought to mirror infiltration by inflammatory cells that contribute to tumor advancement [58]. Moreover, MT1-MMP has been detected in exosomes shed by both tumor cells and stromal fibroblasts, where it functions as a key mediator of latent MMP-2 activation and contributes to the breakdown of ECM proteins [59]. Consistent with these observations, our study demonstrated a similar pattern in plasma-derived L-EVs from pLNM patients, where active MMP-2 and latent MMP-9 were the predominant proteolytic forms, a pattern that aligns with the IHC data from HPA showing moderate MMP-2 expression in tumor cells but undetectable MMP-9 levels in both normal and malignant tissues.

However, the literature on circulating MMP levels in breast cancer, particularly in relation to nodal status, has been inconsistent. Previous studies have associated elevated serum MMP-2 and MMP-9 levels with LNM, with MMP-2 and MMP-9 activity observed only in metastatic LNs [60,61], whereas others reported no significant association between LNM and MMP-2 or MMP-9 expression or activity [62,63]. Plasma MMP-8 has also shown a more complex pattern, correlating positively with LNM while inversely relating to the risk of distant metastasis [64]. One plausible explanation for these inconsistencies is the methodological and biological heterogeneity among studies, including variation in biospecimen type (serum versus plasma), the specific analyte measured (total versus active forms), and the pre-analytical release of MMPs from blood cells during coagulation, particularly in the case of MMP-9 [65,66]. In the present study, we examined MMPs specifically in plasma-derived L-EVs rather than measuring total MMP levels in whole circulation. Because EV cargo is selectively loaded during vesicle biogenesis and circulating EVs participate in tumor, stromal, and immune-cell communication relevant to metastatic progression [67], EV-associated MMP profiling may reflect a biologically distinct compartment that is not necessarily mirrored by total circulating MMP levels.

To further assess clinical relevance, we evaluated associations between MMP transcript expression and survival outcome. In nLNM patients, increased *MMP-8* mRNA was significantly related to reduced OS, while in both nLNM and pLNM groups, it was associated with shorter DMFS and RFS. Similarly, elevated *MMP-9* mRNA expression corresponded to decreased DMFS and RFS in both cohorts, suggesting that *MMP-8* and *MMP-9* may help identify high-risk individuals even in early-stage disease. By contrast, *MMP-2* expression did not demonstrate significant associations with OS, DMFS, or RFS, indicating minimal prognostic relevance in this cohort.

Additionally, analysis of transcriptomic profiles from the ROC plotter database revealed distinct associations between MMP expression and chemotherapy response in breast cancer patients stratified by nodal status. *MMP-2* serves as a consistent indicator of chemotherapy resistance across nodal subgroups, as *MMP-2* was significantly elevated in non-responders in both nLNM and pLNM patients, with ROC analysis indicating modest predictive performance (AUC = 0.565 for nLNM; AUC = 0.571 for pLNM). Higher expression was associated with resistance to FEC therapy in nLNM patients and to anthracycline and taxane regimens in pLNM patients. *MMP-8* retains predictive value primarily in early-stage disease, as *MMP-8* showed increased expression in non-responders with moderate predictive value (AUC = 0.692), particularly for anthracycline treatment, but no significant associations were observed in pLNM patients. In contrast, *MMP-9* did not differ between responders and non-responders in nLNM patients, indicating limited predictive relevance at this stage. However, in pLNM patients, higher *MMP-9* expression was unexpectedly associated with improved response to FEC therapy, suggesting a distinct functional role in advanced nodal disease. Collectively, these findings confirm the utility of *MMP-8* and *MMP-9* as prognostic markers in breast cancer, particularly within high-risk subgroups, and of *MMP-8* and *MMP-2* as indicators of chemotherapy resistance, highlighting their clinical relevance for prognosis and treatment tailoring.

A key limitation of this work is the absence of direct mechanistic data clarifying the cellular source, trafficking, and activation of MMPs within L-EVs. Future studies incorporating functional assays, such as EV uptake by recipient cells and ECM remodeling analyses, will be essential to establish direct functional relevance and further define the biological role of EV-associated MMPs in metastatic progression. In addition, the cohort was limited to chemotherapy-naïve breast cancer patients to maintain sample uniformity and to reduce treatment-related confounding effects on EV composition. Therefore, given the relatively small cohort size and the substantial representation of stage III disease within the pLNM group, some of the observed EV-associated differences may reflect overall disease burden rather than nodal involvement alone. Further, sample pooling was performed to obtain sufficient protein yield for downstream analyses due to the low protein content of L-EV fractions. While pooling enabled reliable detection of EV-associated cargo, it may have masked inter-patient variability and reduced the statistical power of our comparisons because the number of independent biological observations was substantially lower than the number of patients enrolled in each group. Thus, the measured signals reflect average group-level profiles and do not capture patient-to-patient variability. Accordingly, these findings should be interpreted as exploratory group-level observations rather than as definitive patient-level inferences and their potential clinical relevance should be validated in a larger, prospective, and stage-balanced cohort.

## 5. Conclusions

Our results indicate that the latent form of MMP-8, MMP-9, and active MMP-2 were increased in plasma-derived L-EVs from chemotherapy-naïve breast cancer patients with pLNM. Elevated *MMP-8* and *MMP-9* expressions were associated with unfavorable survival outcomes, whereas *MMP-2* and *MMP-8* levels were linked to diminished chemotherapy responsiveness. These findings indicate that MMP-8, along with MMP-2 and MMP-9, may serve as clinically relevant potential non-invasive markers in L-EVs for monitoring breast cancer progression and stratifying patients likely to benefit from specific therapeutic regimens.

## Figures and Tables

**Table 1:** Clinicopathological features of breast cancer patients stratified by lymph node metastasis status (nLNM vs. pLNM).

Characteristic	nLNM (n = 40)	pLNM (n = 32)	*p*-Value
Age (years)			
Range	34–76	36–79	^a^ *p* > 0.05
Mean ± SEM	57.92 ± 2.002	57.69 ± 1.852	
<50	12 (30%)	8 (25%)	^b^ *p* > 0.05
≥50	26 (65%)	24 (75%)	
NA	2 (5%)	-	
**Menopause status, n (%)**			
Premenopausal	14 (35%)	10 (31.25%)	^b^ *p* > 0.05
Postmenopausal	22 (55%)	17 (53.125%)	
NA	4 (10%)	5 (15.625%)	
**Laterality** **, n (%)**			
Bilateral	2 (5%)	1 (3.125%)	^b^ *p* > 0.05
Right	17 (42.5%)	17 (53.125%)	
Left	21 (52.5%)	14 (43.75%)	
**Tumor size (cm), n (%)**			
≤4	34 (85%)	28 (87.5%)	^b^ *p* > 0.05
>4	6 (15%)	4 (12.5%)	
**Tumor grade, n (%)**			
Grade 1	4 (10%)	-	^b^ *p* > 0.05
Grade 2	30 (75%)	27 (84.375%)	
Grade 3	5 (12.5%)	5 (15.625%)	
NA	1 (2.5%)	-	
**ER, n (%)**			
Negative	5 (12.5%)	1 (3.125%)	^b^ *p* > 0.05
Positive	35 (87.5%)	31 (96.875%)	
**PR, n (%)**			
Negative	7 (17.5%)	4 (12.5%)	^b^ *p* > 0.05
Positive	33 (82.5%)	28 (87.5%)	
**HER2, n (%)**			
Negative	34 (85%)	26 (81.25%)	^b^ *p* > 0.05
Equivocal (non-amplified)	6 (15%)	6 (18.75%)	
**Stages, n (%)**			
I	23 (57.5%)	1 (3.125%)	^b^ *p* < 0.05
II	17 (42.5%)	15 (46.875%)	
III	-	16 (50%)	
**Lymph node status, n (%)**			
N0	40 (100%)	-	^b^ *p* < 0.05
N1	-	18 (56.25%)	
N2	-	8 (25%)	
N3	-	6 (18.75%)	
**Tumor size, n (%)**			
T1	23 (57.5%)	8 (25%)	^b^ *p* > 0.05
T2	14 (35%)	22 (68.75%)	
T3	3 (7.5%)	1 (3.125%)	
T4	-	1 (3.125%)	

^a^ *p*-value for one-way ANOVA test. ^b^ *p*-value for Chi-square test.

**Table 2:** Protease array coordinates, characteristics, fold change and *p*-values.

Coordinates	Protein Name	Gene Symbol	Vesiclepedia	Fold Change	*p*-Value	
A11, A12	Cathepsin A	CTSA	√*	0.2	0.030	*
B3, B4	Cathepsin E	CTSE	√	0.2	0.025	*
C5, C6	Kallikrein 7	KLK7	√*	0.4	0.027	*
C7, C8	Kallikrein 10	KLK7	√*	0.3	0.011	*
C11, C12	Kallikrein 13	KLK13	√*	0.5	0.014	*
C13, C14	Matrix metallopeptidase 1	MMP1	√*	0.4	0.011	*
D5, D6	Matrix metallopeptidase 8	MMP8	√*	2.7	0.001	***
D7, D8	Matrix metallopeptidase 9	MMP9	√*	1.5	0.023	*

√* Documented in the Vesiclepedia database for Homo sapiens (http://www.microvesicles.org) (accessed on 1 January 2026). √ Documented in the Vesiclepedia database for species other than Homo sapiens. * *p* < 0.05; *** *p* < 0.001. *p*-values were determined using Student’s *t*-test.

**Table 3:** Correlation of *MMP-2*, *MMP-8* and *MMP-*9 expressions with response to various chemotherapeutic regimens in breast cancer patients.

Gene	Parameter	nLNM				pLNM			
		FEC	Anthracycline	FAC	Taxane	FEC	Anthracycline	FAC	Taxane
***MMP-2***	Responder (expression)	**1178 ± 1398**	1695 ± 1846	2268 ± 2839	1497 ± 1830	1317 ± 1288	**1406 ± 1768**	3326 ± 3303	**1102 ± 110**
		**(n = 32)**	(n = 86)	(n = 18)	(n = 75)	(n = 51)	**(n = 129)**	(n = 14)	**(n = 110)**
	Non-responder (expression)	**1691 ± 1281 (n = 56)**	2187 ± 2263	2690 ± 3030	1738 ± 2016	1567 ± 1365	**1688 ± 1890**	1400 ± 1736	**1452 ± 1736**
			(n = 276)	(n = 51)	(n = 196)	(n = 100)	**(n = 483)**	(n = 91)	**(n = 379)**
	ROC *p*-value	**4.30 × 10^−3^**	0.058	0.47	0.2	0.21	**7.30 × 10^−3^**	0.077	**2.60 × 10^−3^**
	AUC	**0.665**	0.554	0.507	0.533	0.539	**0.57**	0.639	**0.586**
	Mann–Whitney test *p*-value	**0.011**	0.13	0.94	0.4	0.43	**0.014**	0.096	**0.0058**
	Fold change	**1.4**	1.3	1.2	1.2	1.2	**1.2**	2.4	**1.3**
***MMP-8***	Responder (expression)	16 ± 13	**12 ± 10**	0	16 ± 13	12 ± 10	25 ± 42	0	12 ± 10
		(n = 4)	**(n = 10)**		(n = 4)	(n = 23)	(n = 33)		(n = 23)
	Non-responder (expression)	10 ± 7	**30 ± 42**	0	10 ± 7	23 ± 93	31 ±77	0	23 ± 93
		(n = 28)	**(n = 74)**		(n = 28)	(n = 60)	(n = 112)		(n = 60)
	ROC *p*-value	0.25	**9.00 × 10^−3^**		0.25	0.28	0.42		0.28
	AUC	0.621	**0.692**		0.621	0.541	0.511		0.541
	Mann–Whitney test *p*-value	0.46	**0.051**		0.46	0.57	0.85		0.57
	Fold change	1.6	**2.5**		1.6	2	1.2		2
***MMP-9***	Responder (expression)	2083 ± 1908	3338 ± 6489	6154 ± 13,238 (n = 18)	3301 ± 6901 (n = 75)	**2319 ± 3894**	2777 ± 3761	3570 ± 5513	2620 ± 3546
		(n = 32)	(n = 86)			**(n = 51)**	(n = 129)	(n = 14)	(n = 110)
	Non-responder (expression)	3164 ± 6229	3203 ± 5459	3332 ± 5068	3310 ± 5928 (n = 196)	**1437 ± 1962**	2665 ± 5170	2350 ± 2526	2614 ± 5614
		(n = 56)	(n = 276)	(n = 51)		**(n = 100)**	(n = 483)	(n = 91)	(n = 379)
	ROC *p*-value	0.1	0.22	0.15	0.3	**3.00 × 10^−3^**	0.23	0.11	0.27
	AUC	0.577	0.527	0.58	0.52	**0.629**	0.521	0.591	0.519
	Mann–Whitney test *p*-value	0.23	0.45	0.32	0.61	**0.0099**	0.46	0.28	0.54
	Fold change	1.5	1	1.8	1	**1.6**	1	1.5	1

Bold values indicate statistically significant *p*-values.

## Data Availability

The datasets generated during and/or analyzed during the current study are available from the corresponding author upon reasonable request.

## Supplementary Materials

The following supporting information can be downloaded at: https://www.mdpi.com/article/10.3390/cancers18091464/s1, Figure S1: Raw data for Figure 1a. Full size of Transmission Electron Microscopy (TEM) image of representative independent sample; Figure S2: Raw data for Figure 1a. Full size of Transmission Electron Microscopy (TEM) image of representative independent sample; Figure S3: Raw data for Figure 1a. Full size of Transmission Electron Microscopy (TEM) image of representative independent sample; Figure S4: Raw data for Figure 1a. Full size of Transmission Electron Microscopy (TEM) image of representative independent sample; Figure S5: Raw data for Figure 1c. First original dot blot image of EV marker CD9 in independently pooled L-EV samples from each group (nLNM: EV1 (n = 10) and EV2 (n = 10); pLNM: EV1 (n = 8) and EV2 (n = 8)), corresponding post-pelleting supernatants (S.N1 and S.N2) and a cocktail of total human cell line lysates (+Ve C); Figure S6: Raw data for Figure 1c. Second original dot blot image of EV marker CD9 in different independently pooled L-EV samples from each group (nLNM: EV1 (n = 10) and EV2 (n = 10); pLNM: EV1 (n = 8) and EV2 (n = 8)), corresponding post-pelleting supernatants (S.N1 and S.N2) and a cocktail of total human cell line lysates (+Ve C); Figure S7: Raw data for Figure 1c. First original dot blot image of EV marker HSP70 in independently pooled L-EV samples from each group (nLNM: EV1 (n = 10) and EV2 (n = 10); pLNM: EV1 (n = 8) and EV2 (n = 8)), corresponding post-pelleting supernatants (S.N1 and S.N2) and a cocktail of total human cell line lysates (+Ve C); Figure S8: Raw data for Figure 1c. Second original dot blot image of EV marker HSP70 in different independently pooled L-EV samples from each group (nLNM: EV1 (n = 10) and EV2 (n = 10); pLNM: EV1 (n = 8) and EV2 (n = 8)), corresponding post-pelleting supernatants (S.N1 and S.N2) and a cocktail of total human cell line lysates (+Ve C); Figure S9: Raw data for Figure 1c. First original dot blot image of EV marker ALIX in independently pooled L-EV samples from each group (nLNM: EV1 (n = 10) and EV2 (n = 10); pLNM: EV1 (n = 8) and EV2 (n = 8)), corresponding post-pelleting supernatants (S.N1 and S.N2) and a cocktail of total human cell lines lysate (+Ve C).; Figure S10: Raw data for Figure 1c. Second original dot blot image of EV marker ALIX in different independently pooled L-EV samples from each group (nLNM: EV1 (n = 10) and EV2 (n = 10); pLNM: EV1 (n = 8) and EV2 (n = 8)), corresponding post-pelleting supernatants (S.N1 and S.N2) and a cocktail of total human cell line lysates (+Ve C); Figure S11: Raw data for Figure 1d. First original dot blot image of non-EV marker Calnexin in independently pooled L-EV samples from each group (nLNM: EV1 (n = 10) and EV2 (n = 10); pLNM: EV1 (n = 8) and EV2 (n = 8)), corresponding post-pelleting supernatants (S.N1 and S.N2) and a cocktail of total human cell line lysates (+Ve C); Figure S12: Raw data for Figure 1d. Original image of Ponceau S stain of the corresponding membrane as a total protein loading in independently pooled L-EV samples from each group (nLNM: EV1 (n = 10) and EV2 (n = 10); pLNM: EV1 (n = 8) and EV2 (n = 8)), corresponding post-pelleting supernatants (S.N1 and S.N2) and a cocktail of total human cell line lysates (+Ve C); Figure S13: Raw data for Figure 1d. Second original dot blot image of non-EV marker Calnexin in different independently pooled L-EV samples from each group (nLNM: EV1 (n = 10) and EV2 (n = 10); pLNM: EV1 (n = 8) and EV2 (n = 8)), corresponding post-pelleting supernatants (S.N1 and S.N2) and a cocktail of total human cell line lysates (+Ve C); Figure S14: Raw data for Figure 1e. First original Western blot image of EV marker CD9 in independently pooled L-EV samples from each group (nLNM: EV1 (n = 6), EV2 (n = 7) and EV3 (n = 7); pLNM: EV1 (n = 5), EV2 (n = 5) and EV3 (n = 6)); Figure S15: Raw data for Figure 1e. Second original Western blot image of EV marker CD9 in different independently pooled L-EV samples from each group (nLNM: EV1 (n = 6), EV2 (n = 7) and EV3 (n = 7); pLNM: EV1 (n = 5), EV2 (n = 5) and EV3 (n = 6)); Figure S16: Raw data for Figure 2a. First original protease array image of pooled L-EV samples from each group (nLNM: n = 5; pLNM: n = 5); Figure S17: Raw data for Figure 2a. Second Original protease array image of different pooled L-EV samples from each group (nLNM: n = 5; pLNM: n = 5); Figure S18: Raw data for Figure 3a. First original Western blot image of L-EV-derived MMP-8 using independently prepared pooled L-EV samples from each group (nLNM: EV1 (n = 4), EV2 (n = 5) and EV3 (n = 5); pLNM: EV1 (n = 3), EV2 (n = 3) and EV3 (n = 4)); Figure S19: Raw data for Figure 3a. Second original Western blot image of L-EV-derived MMP-8 using different independently prepared pooled L-EV samples from each group (nLNM: EV1 (n = 4), EV2 (n = 4) and EV3 (n = 5); pLNM: EV1 (n = 3), EV2 (n = 4) and EV3 (n = 4)); Figure S20: Raw data for Figure 3a. Third original Western blot image of L-EV-derived MMP-8 using different independently prepared pooled L-EV samples from each group (nLNM: EV1 (n = 4), EV2 (n = 4) and EV3 (n = 5); pLNM: EV1 (n = 3), EV2 (n = 4) and EV3 (n = 4)); Figure S21: Raw data for Figure 3a. Original Western blot image of L-EV-derived CD9 using independently prepared pooled L-EV samples from each group (nLNM: EV1 (n = 4), EV2 (n = 5) and EV3 (n = 5); pLNM: EV1 (n = 3), EV2 (n = 3) and EV3 (n = 4)); Figure S22: Raw data for Figure 3c. First original Western blot image of tumor-derived MMP-8 in nLNM (n = 4) and pLNM (n = 4) patients; Figure S23: Raw data for Figure 3c. First original Western blot image of tumor-derived β-actin in nLNM (n = 4) and pLNM (n = 4) patients; Figure S24: Raw data for Figure 3c. First original image of Ponceau S stain of the corresponding membrane as a total protein loading in nLNM (n = 4) and pLNM (n = 4) patients; Figure S25: Raw data for Figure 3c. Second original Western blot image of tumor-derived MMP-8 in different nLNM (n = 4) and pLNM (n = 4) patients; Figure S26: Raw data for Figure 3c. Second original Western blot image of tumor-derived β-actin in different nLNM (n = 4) and pLNM (n = 4) patients; Figure S27: Raw data for Figure 3c. Second original image of Ponceau S stain of the corresponding membrane as a total protein loading in different nLNM (n = 4) and pLNM (n = 4) patients; Figure S28: Raw data for Figure 3c. Third original Western blot image of tumor-derived MMP-8 in different nLNM (n = 4) and pLNM (n = 4) patients; Figure S29: Raw data for Figure 3c. Third original Western blot image of tumor-derived β-actin in different nLNM (n = 4) and pLNM (n = 4) patients; Figure S30: Raw data for Figure 3c. Third original image of Ponceau S stain of the corresponding membrane as a total protein loading in different nLNM (n = 4) and pLNM (n = 4) patients; Figure S31: Raw data for Figure 4a. Original zymography image of plasma samples derived from nLNM (n = 3) and pLNM (n = 5) patients; Figure S32: Raw data for Figure 4c,e. First original zymography image of L-EV samples using independently prepared pooled L-EV samples from each group (nLNM: EV1 (n = 6), EV2 (n = 7) and EV3 (n = 7); pLNM: EV1 (n = 5), EV2 (n = 5) and EV3 (n = 6)) and the corresponding post-pelleting supernatant fraction samples; Figure S33: Raw data for Figure 4c. Second original zymography image of L-EV samples using different independently prepared pooled L-EV samples from each group (nLNM: EV1 (n = 6), EV2 (n = 7) and EV3 (n = 7); pLNM: EV1 (n = 5), EV2 (n = 5) and EV3 (n = 6)); Table S1. List of antibodies used in this study; Table S2. List of primers used in this study.

## Abbreviations

The following abbreviations are used in this manuscript:

LNM	Lymph node metastasis
EVs	Extracellular vesicles
ECM	Extracellular matrix
MMPs	Matrix metalloproteinases
nLNM	Negative LNM
pLNM	Positive LNM
PBS	Phosphate-buffered saline
L-EVs	Large extracellular vesicles
ACD	Acid–citrate–dextrose
PPP	Platelet-poor plasma
RT	Room temperature
ISEV	International Society for Extracellular Vesicles
FSC-A	Forward scatter area
SSC-A	Side scatter area
TEM	Transmission electron microscopy
HR-TEM	High-resolution TEM
DLS	Dynamic light scattering
PDI	Polydispersity index
HRP	Horseradish peroxidase
ECL	Enhanced chemiluminescent
qRT-PCR	Quantitative real-time PCR
TCGA	The Cancer Genome Atlas
IHC	Immunohistochemistry
HPA	Human Protein Atlas
LN	Lymph node
OS	Overall survival
RFS	Relapse-free survival
DMFS	Distant metastasis-free survival
HRs	Hazard ratios
CIs	Confidence intervals
ROC	Receiving operating characteristics
pCR	Pathological complete response
AUC	Area under the curve
ANOVA	One-way analysis of variance
FEC	Fluorouracil, epirubicin, and cyclophosphamide
FAC	Fluorouracil, adriamycin, and cyclophosphamide
TME	Tumor microenvironment
