# Supplementary material for: Large Extracellular Vesicle-Derived Latent MMP-8 and Gelatinolytically Active MMP-2 as Potential Circulating Markers for Lymph Node Metastasis in Breast Cancer
Source: Cancers (Basel). 2026 May 2;18(9):1464. doi: 10.3390/cancers18091464 (PMC13162927; doi:10.3390/cancers18091464)
Supplement: Supplementary file 1 [file cancers-18-01464-s001.zip › cancers-4291713-supplementary.pdf]

cancers

MDPI

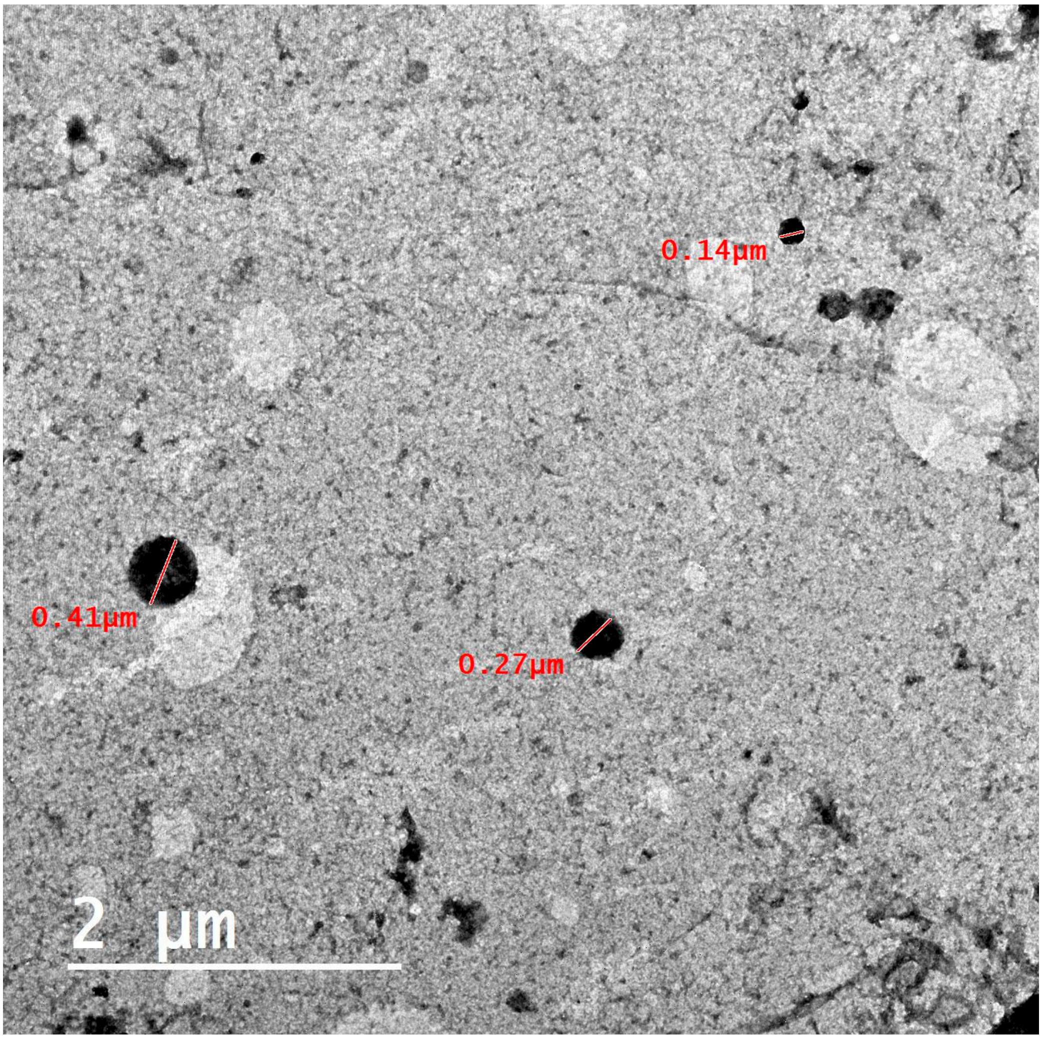

Figure S1. Raw data for Fig. 1a. Full size of Transmission Electron Microscopy (TEM) image of representative independent sample.

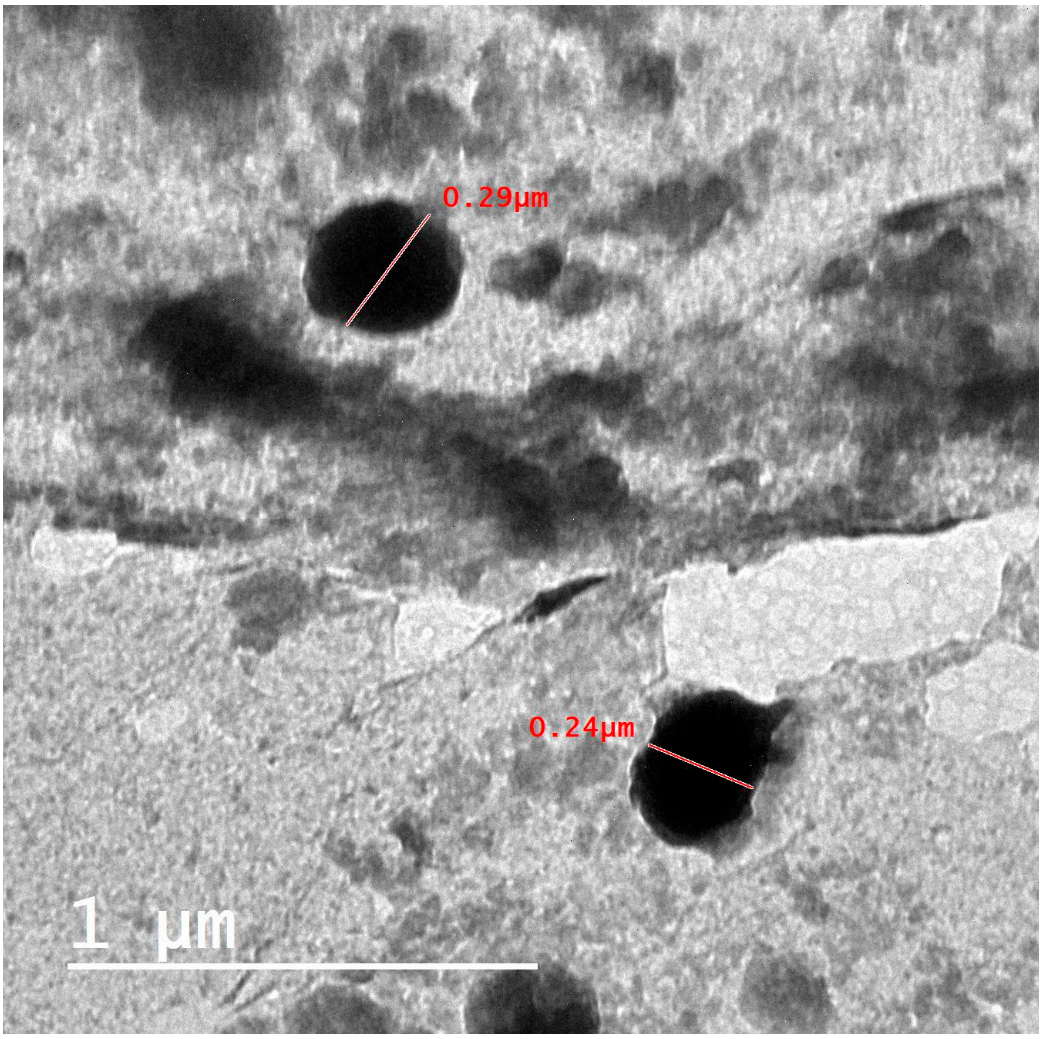

Figure S2. Raw data for Fig. 1a. Full size of Transmission Electron Microscopy (TEM) image of representative independent sample.

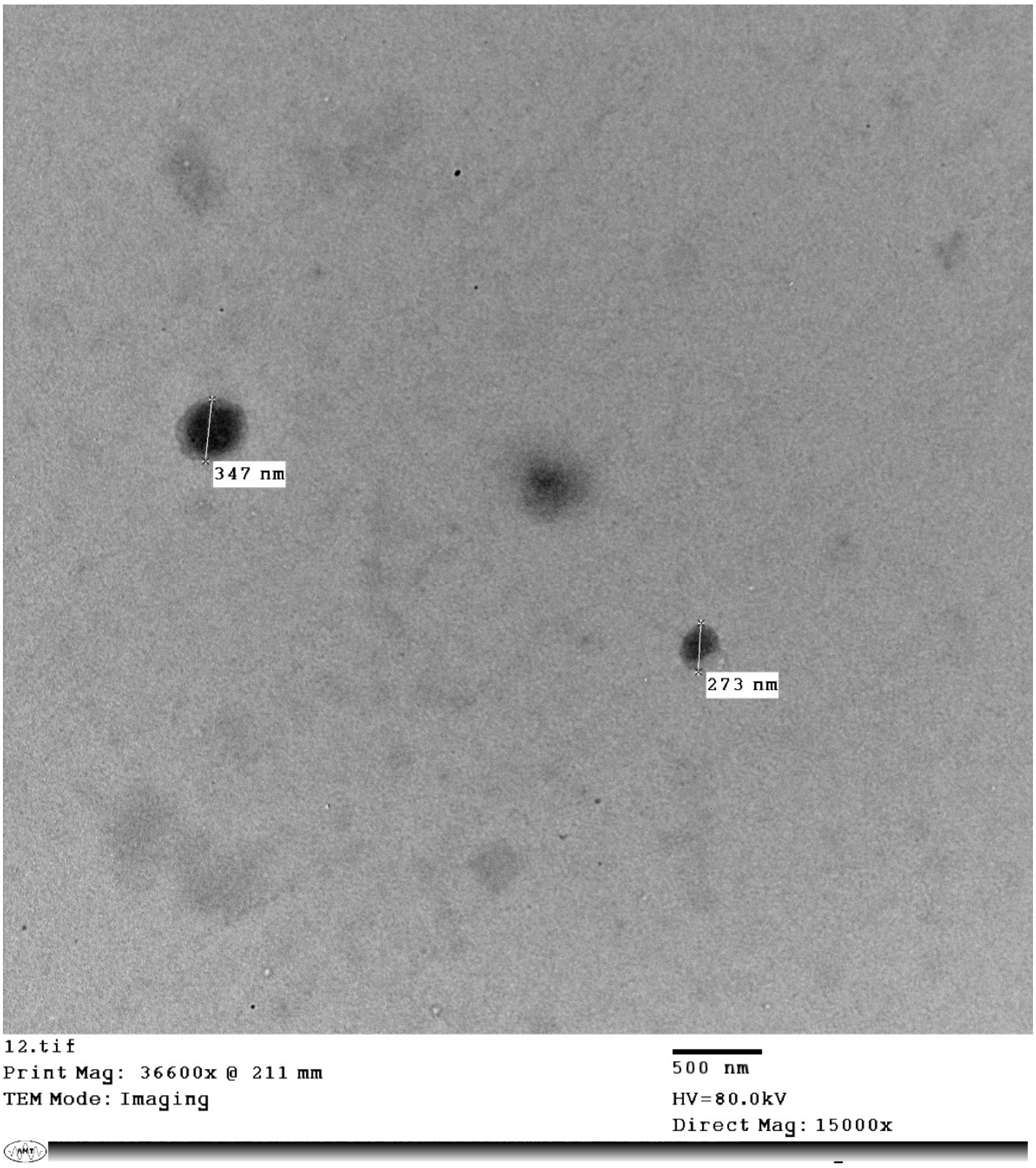

Figure S3. Raw data for Fig. 1a. Full size of Transmission Electron Microscopy (TEM) image of representative independent sample.

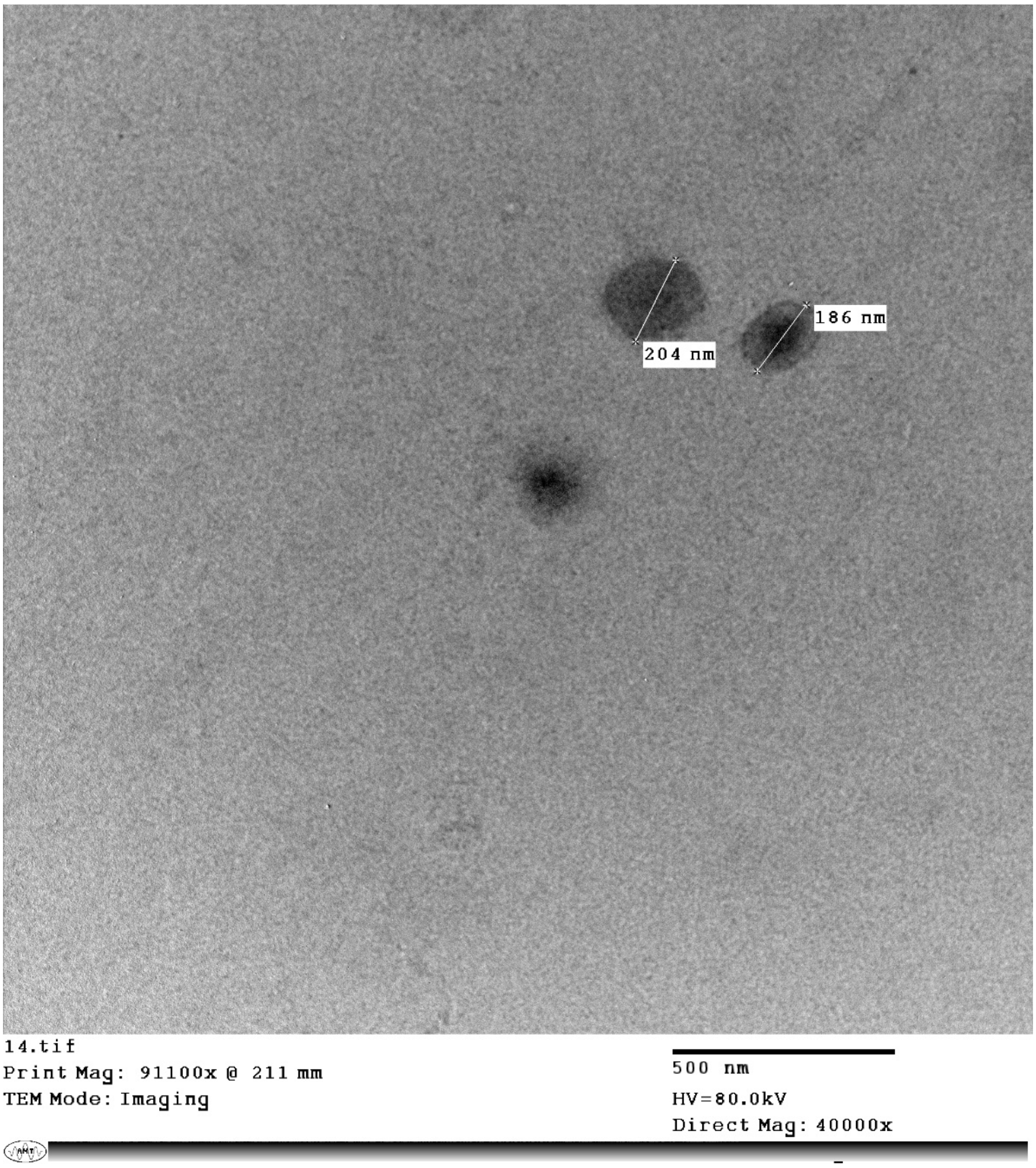

Figure S4. Raw data for Fig. 1a. Full size of Transmission Electron Microscopy (TEM) image of representative independent sample.

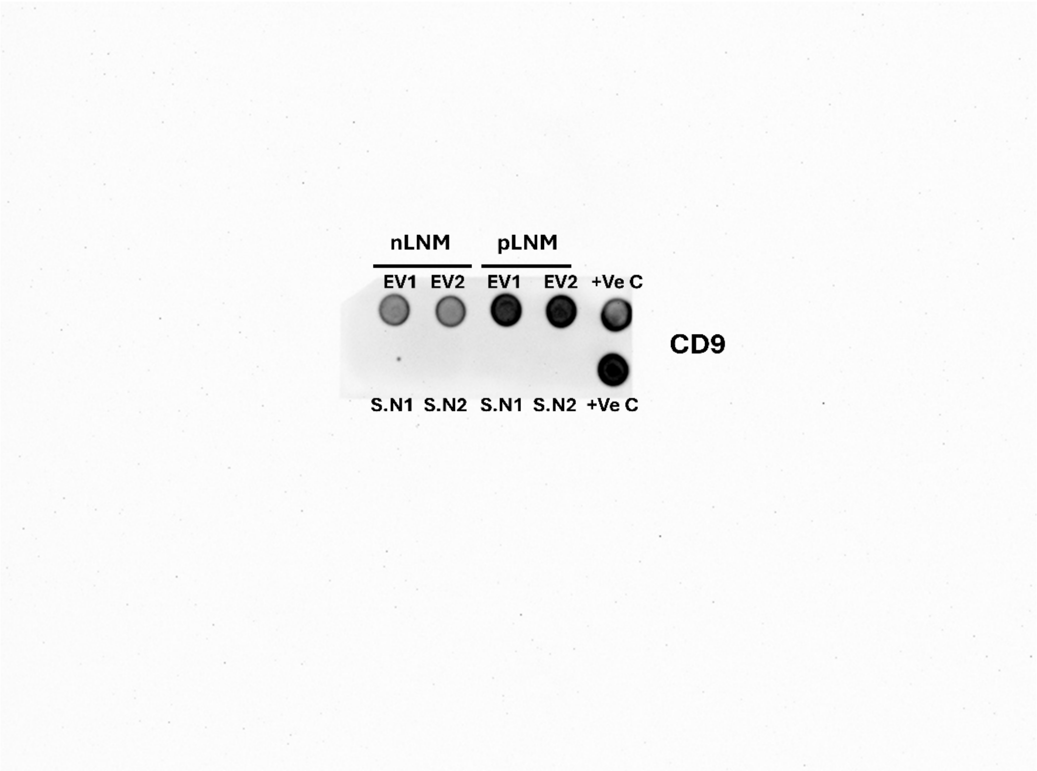

Figure S5. Raw data for Fig. 1c. First original dot blot image of EV marker CD9 in independently pooled L-EV samples from each group (nLNM: EV1 (n = 10) and EV2 (n = 10); pLNM: EV1 (n = 8) and EV2 (n = 8)), corresponding post-pelleting supernatants (S.N1 and S.N2) and a cocktail of total human cell lines lysate (+Ve C).

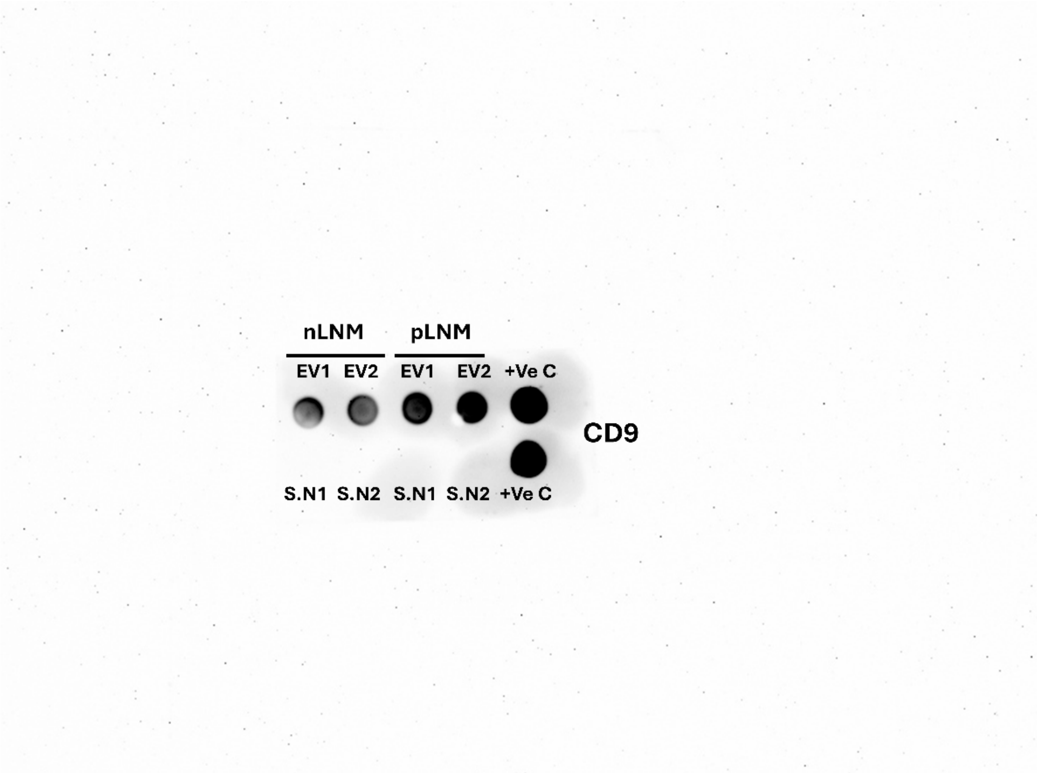

Figure S6. Raw data for Fig. 1c. Second original dot blot image of EV marker CD9 in different independently pooled L-EV samples from each group (nLNM: EV1 (n = 10) and EV2 (n = 10); pLNM: EV1 (n = 8) and EV2 (n = 8)), corresponding post-pelleting supernatants (S.N1 and S.N2) and a cocktail of total human cell lines lysate (+Ve C).

https://doi.org/10.3390/cancers18091461

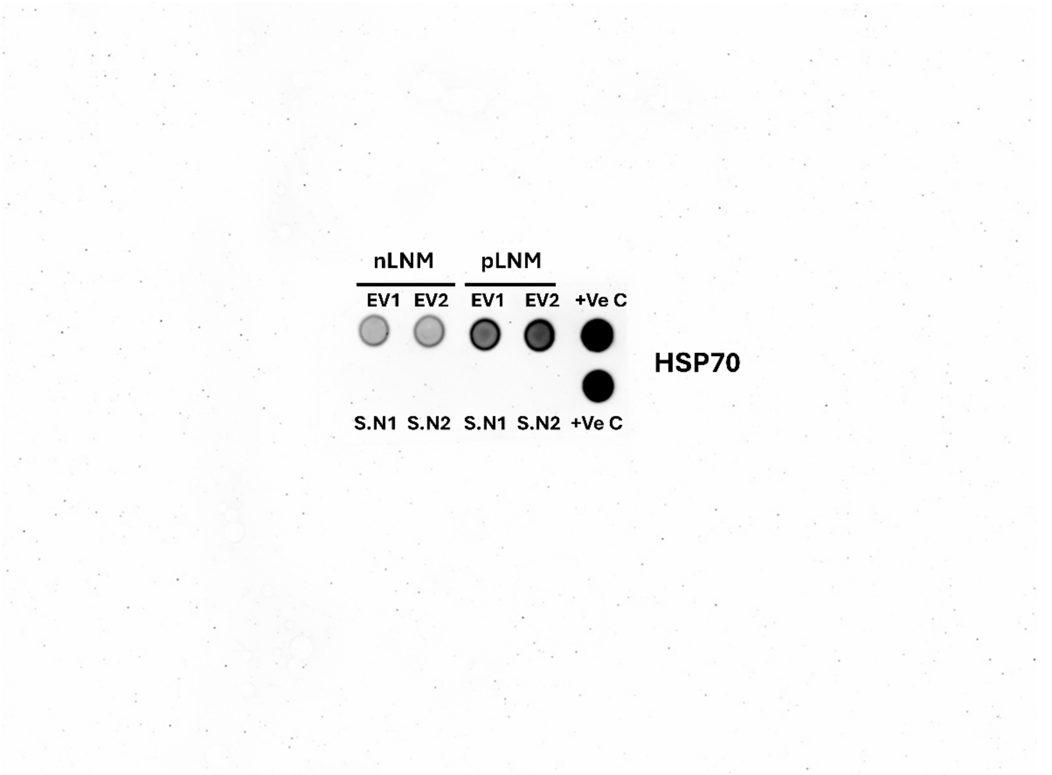

Figure S7. Raw data for Fig. 1c. First original dot blot image of EV marker HSP70 in independently pooled L-EV samples from each group (nLNM: EV1 (n = 10) and EV2 (n = 10); pLNM: EV1 (n = 8) and EV2 (n = 8)), corresponding post-pelleting supernatants (S.N1 and S.N2) and a cocktail of total human cell lines lysate (+Ve C).

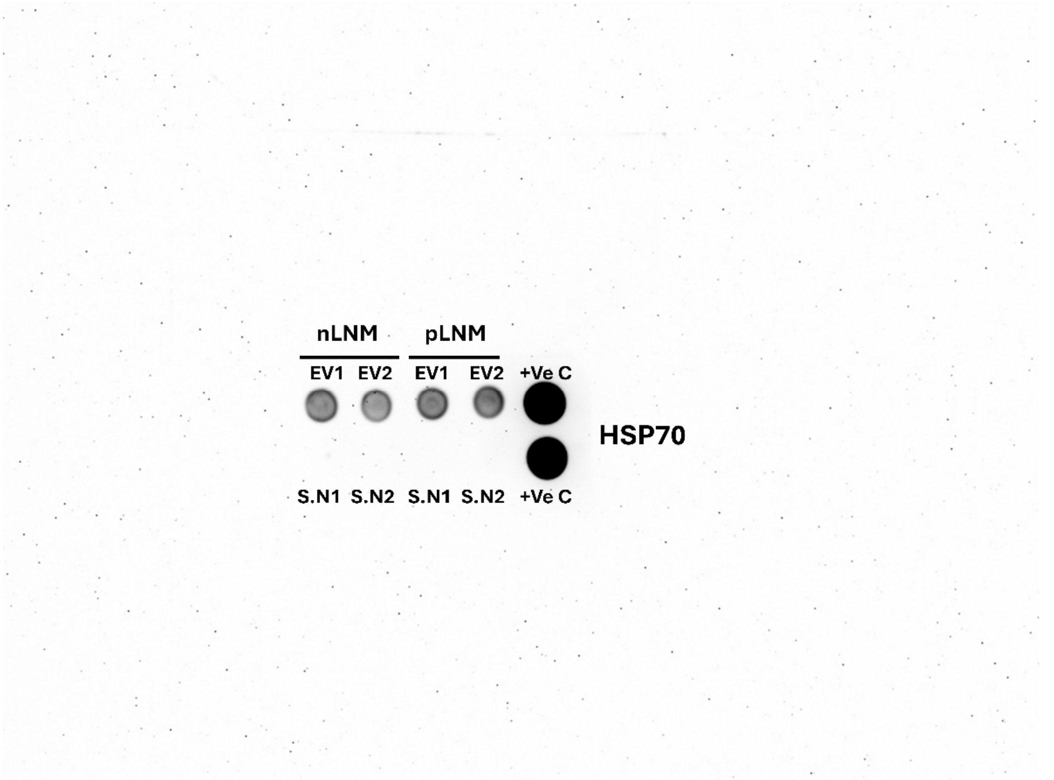

Figure S8. Raw data for Fig. 1c. Second original dot blot image of EV marker HSP70 in different independently pooled L-EV samples from each group (nLNM: EV1 (n = 10) and EV2 (n = 10); pLNM: EV1 (n = 8) and EV2 (n = 8)), corresponding post-pelleting supernatants (S.N1 and S.N2) and a cocktail of total human cell lines lysate (+Ve C).

https://doi.org/10.3390/cancers18091461

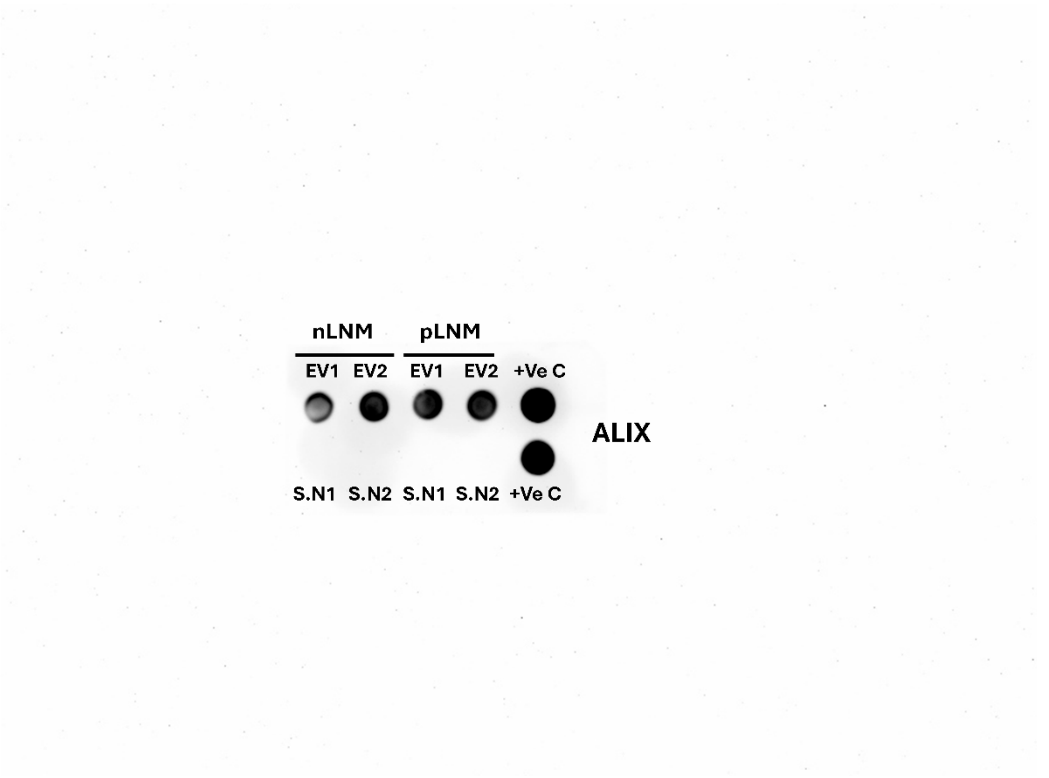

Figure S9. Raw data for Fig. 1c. First original dot blot image of EV marker ALIX in independently pooled L-EV samples from each group (nLNM: EV1 (n = 10) and EV2 (n = 10); pLNM: EV1 (n = 8) and EV2 (n = 8)), corresponding post-pelleting supernatants (S.N1 and S.N2) and a cocktail of total human cell lines lysate (+Ve C).

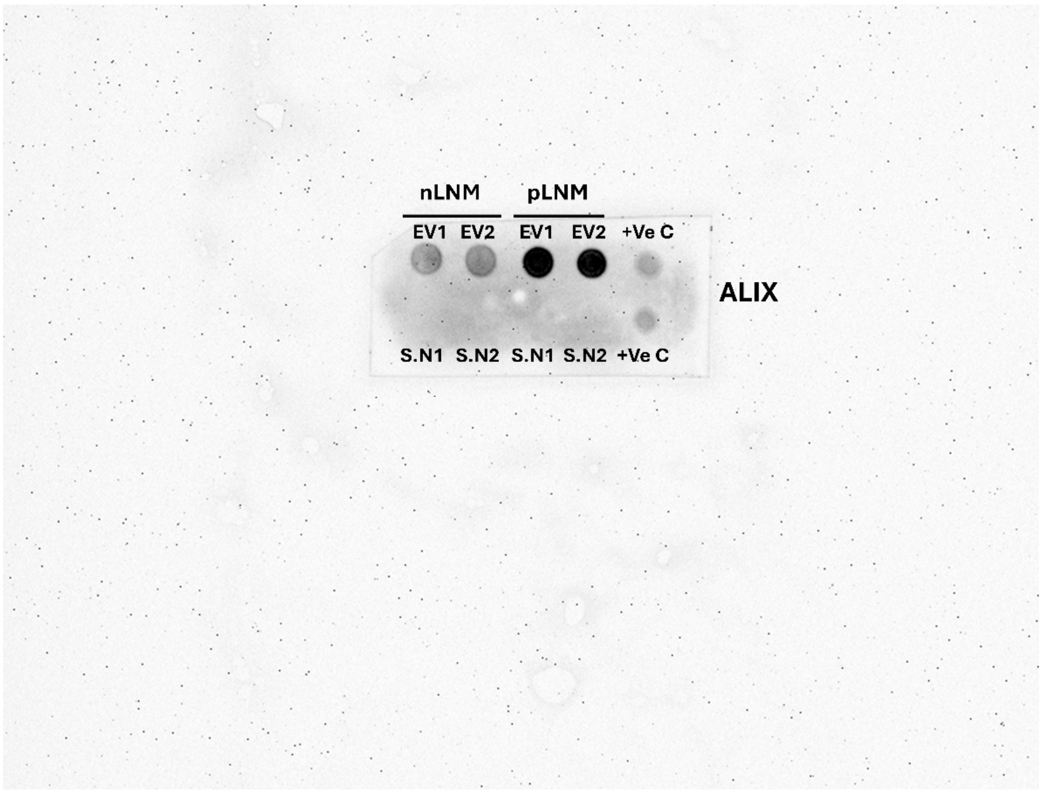

Figure S10. Raw data for Fig. 1c. Second original dot blot image of EV marker ALIX in different independently pooled L-EV samples from each group (nLNM: EV1 (n = 10) and EV2 (n = 10); pLNM: EV1 (n = 8) and EV2 (n = 8)), corresponding post-pelleting supernatants (S.N1 and S.N2) and a cocktail of total human cell lines lysate (+Ve C).

https://doi.org/10.3390/cancers18091461

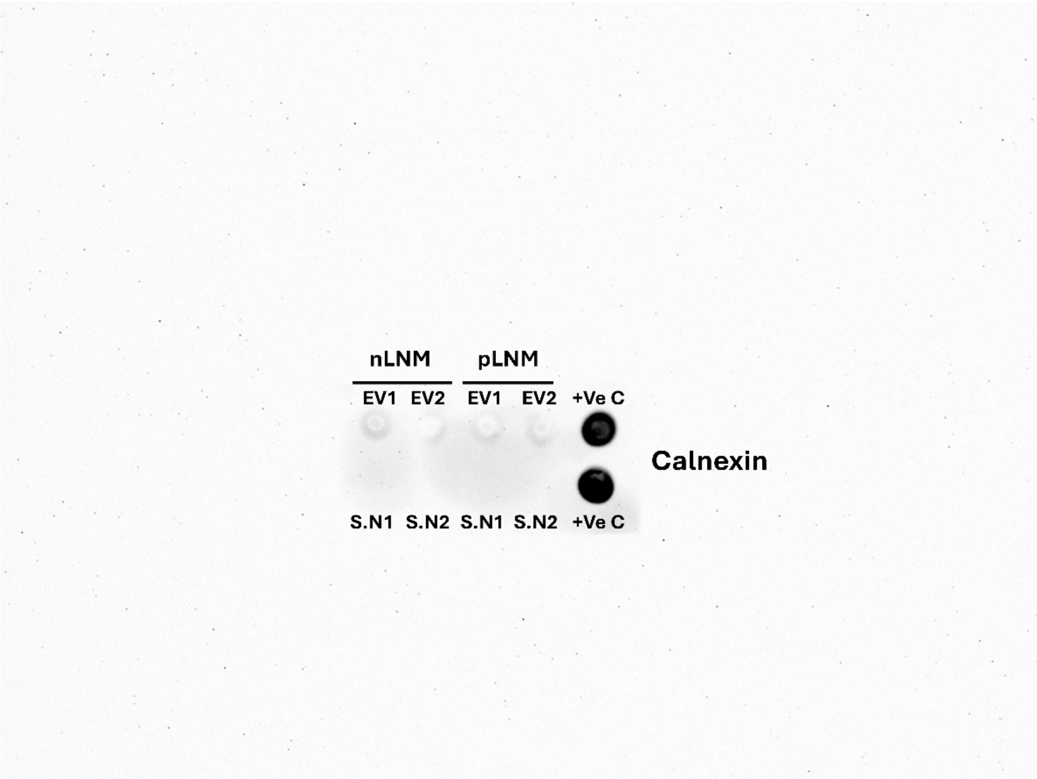

Figure S11. Raw data for Fig. 1d. First original dot blot image of non-EV marker Calnexin in independently pooled L-EV samples from each group (nLNM: EV1 (n = 10) and EV2 (n = 10); pLNM: EV1 (n = 8) and EV2 (n = 8)), corresponding post-pelleting supernatants (S.N1 and S.N2) and a cocktail of total human cell lines lysate (+Ve C).

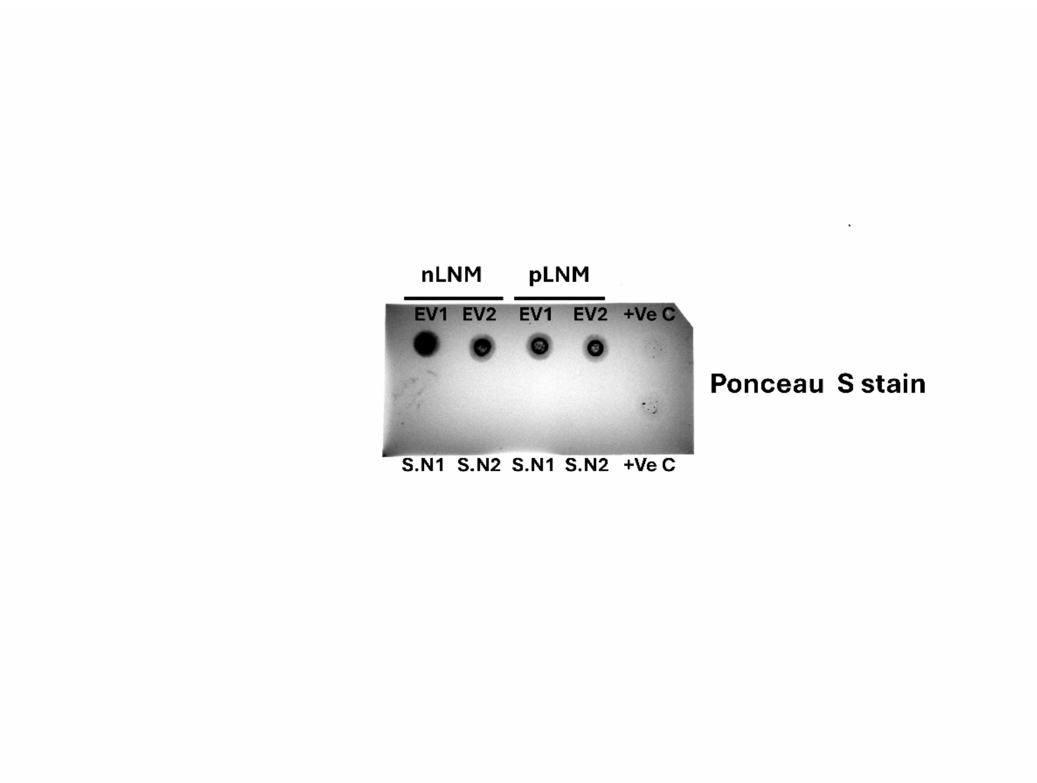

Figure S12. Raw data for Fig. 1d. Original image of Ponceau S stain of the corresponding membrane as a total protein loading in independently pooled L-EV samples from each group (nLNM: EV1 (n = 10) and EV2 (n = 10); pLNM: EV1 (n = 8) and EV2 (n = 8)), corresponding post-pelleting supernatants (S.N1 and S.N2) and a cocktail of total human cell lines lysate (+Ve C).

https://doi.org/10.3390/cancers18091461

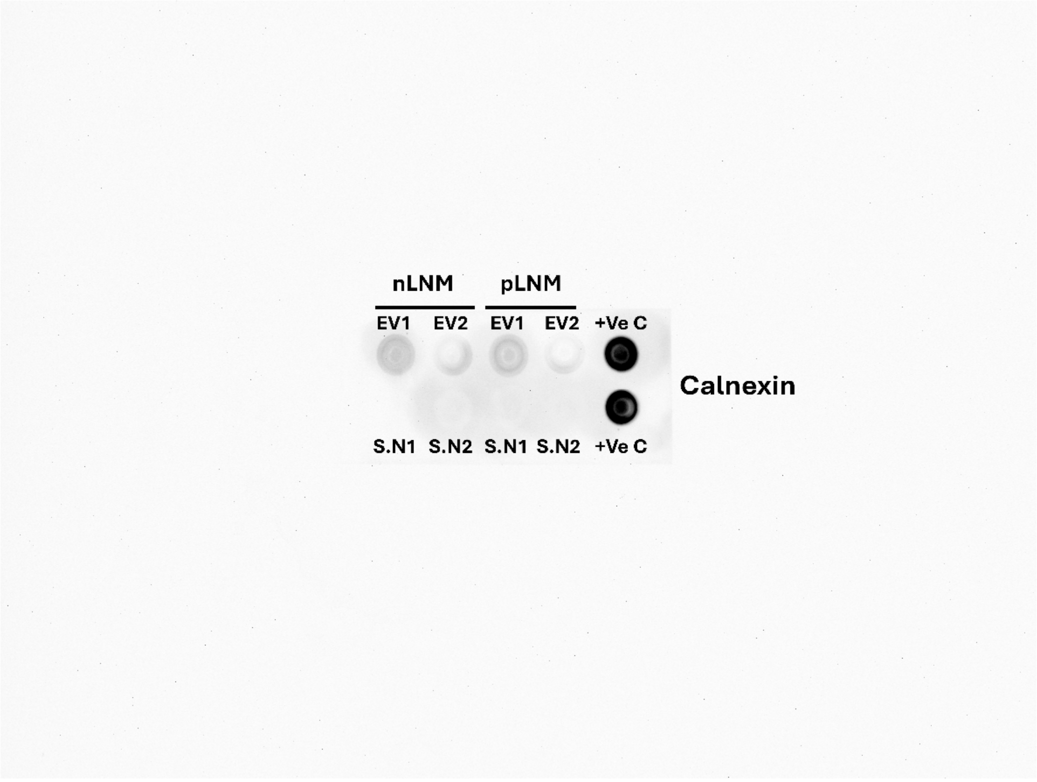

Figure S13. Raw data for Fig. 1d. Second original dot blot image of non-EV marker Calnexin in different independently pooled L-EV samples from each group (nLNM: EV1 (n = 10) and EV2 (n = 10); pLNM: EV1 (n = 8) and EV2 (n = 8)), corresponding post-pelleting supernatants (S.N1 and S.N2) and a cocktail of total human cell lines lysate (+Ve C).

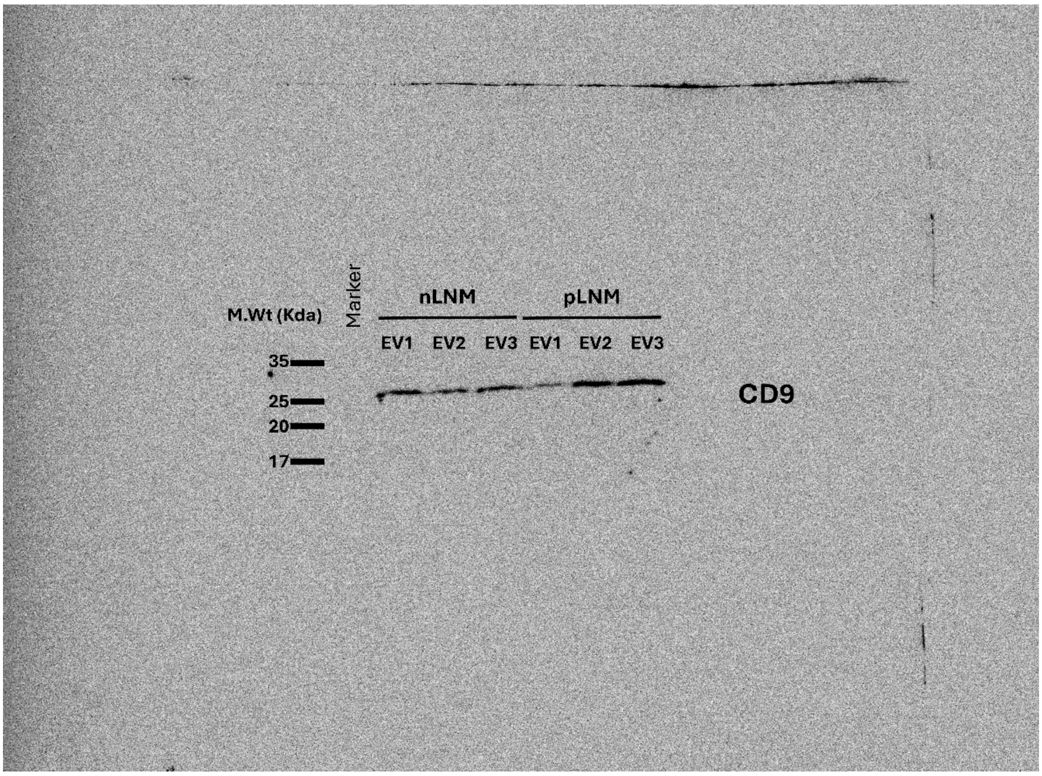

Figure S14. Raw data for Fig. 1e. First original western blot image of EV marker CD9 in independently pooled L-EV samples from each group (nLNM: EV1 (n = 6), EV2 (n = 7) and EV3 (n = 7); pLNM: EV1 (n = 5), EV2 (n = 5) and EV3 (n = 6)).

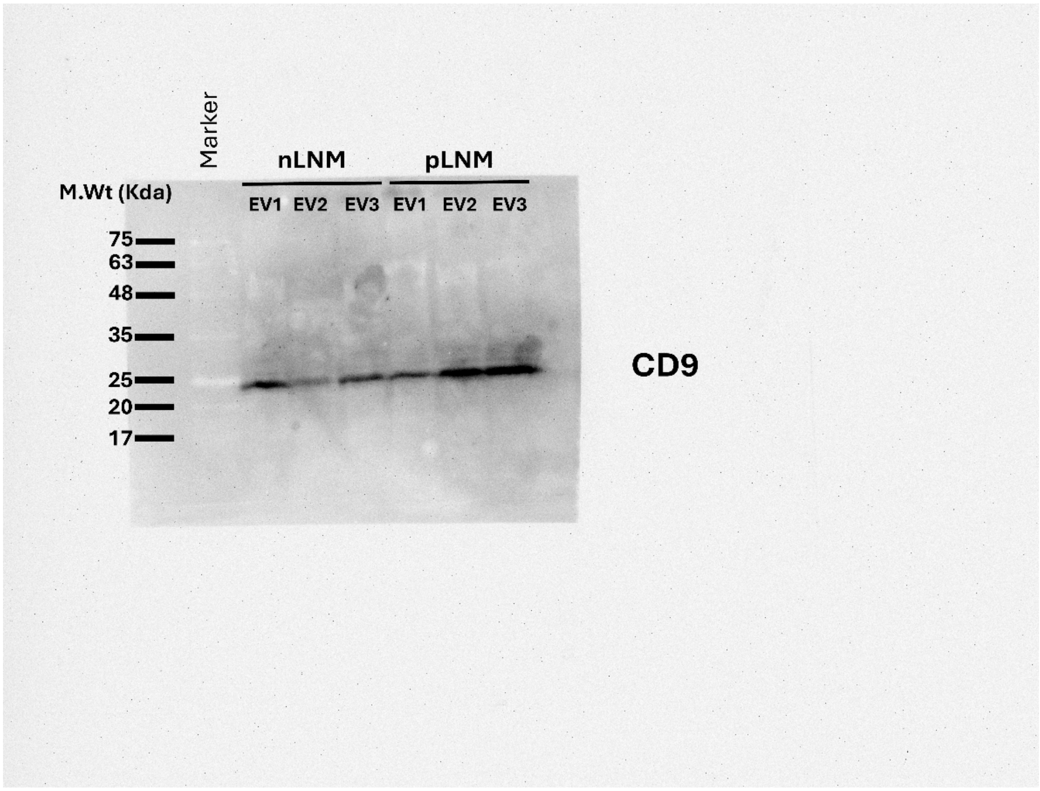

Figure S15. Raw data for Fig. 1e. Second original western blot image of EV marker CD9 in different independently pooled L-EV samples from each group (nLNM: EV1 (n = 6), EV2 (n = 7) and EV3 (n = 7); pLNM: EV1 (n = 5), EV2 (n = 5) and EV3 (n = 6)).

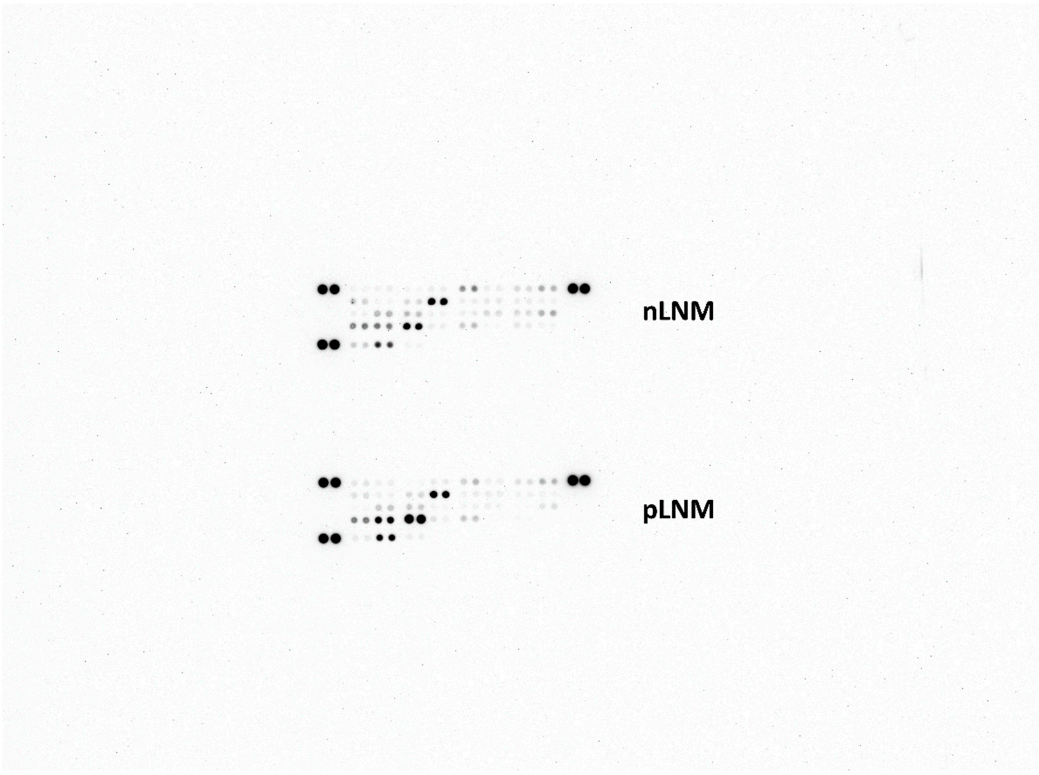

Figure S16. Raw data for Fig. 2a. First original protease array image of pooled L-EV samples from each group (nLNM: n = 5; pLNM: n = 5) .

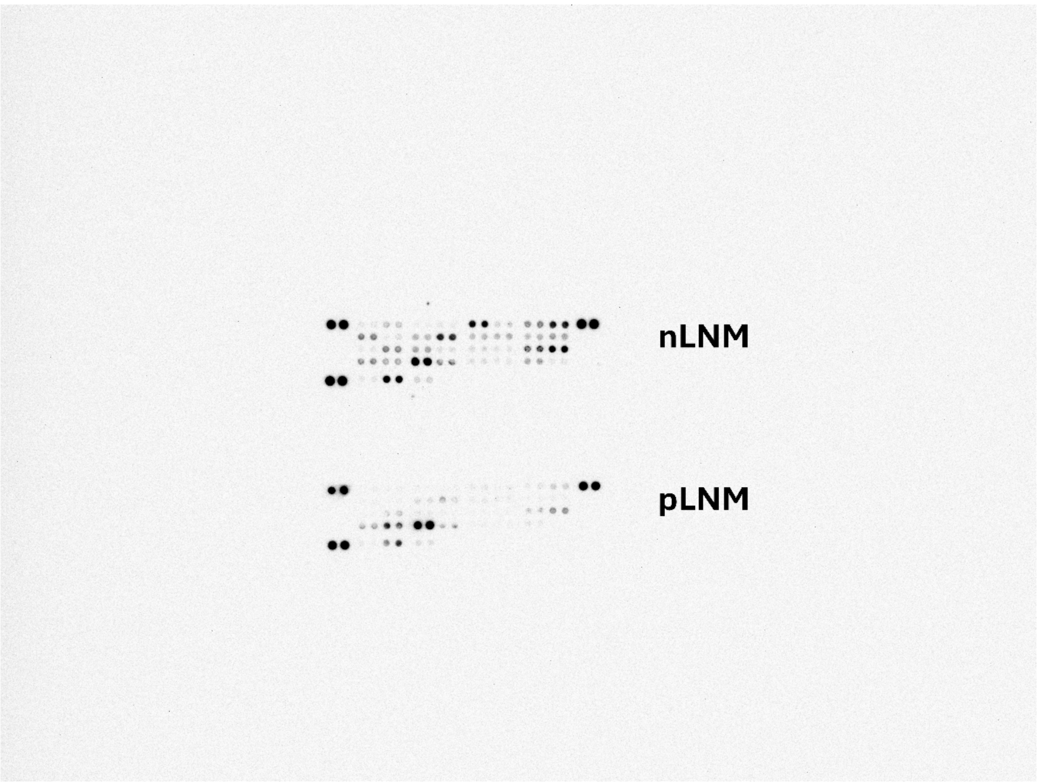

Figure S17. Raw data for Fig. 2a. Second Original protease array image of different pooled L-EV samples from each group (nLNM: n = 5; pLNM: n = 5) .

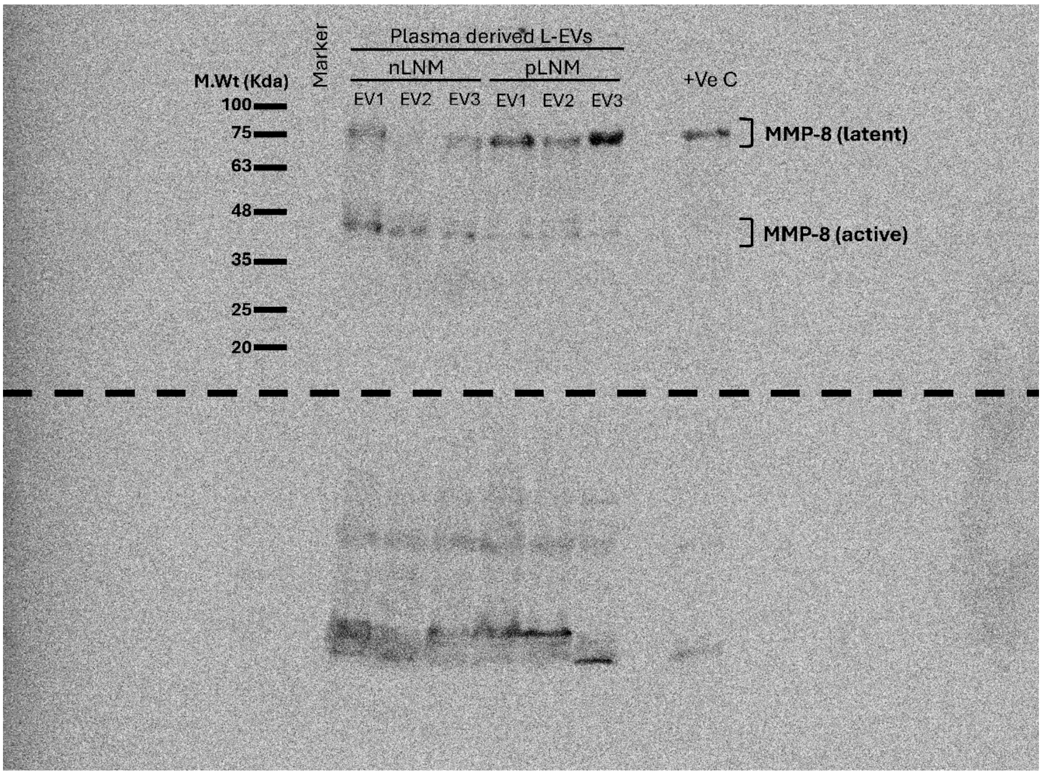

Figure S18. Raw data for Fig. 3a. First original western blot image of L-EV-derived MMP-8 using independently prepared pooled L-EV samples from each group (nLNM: EV1 (n = 4), EV2 (n = 5) and EV3 (n = 5); pLNM: EV1 (n = 3), EV2 (n = 3) and EV3 (n = 4)).

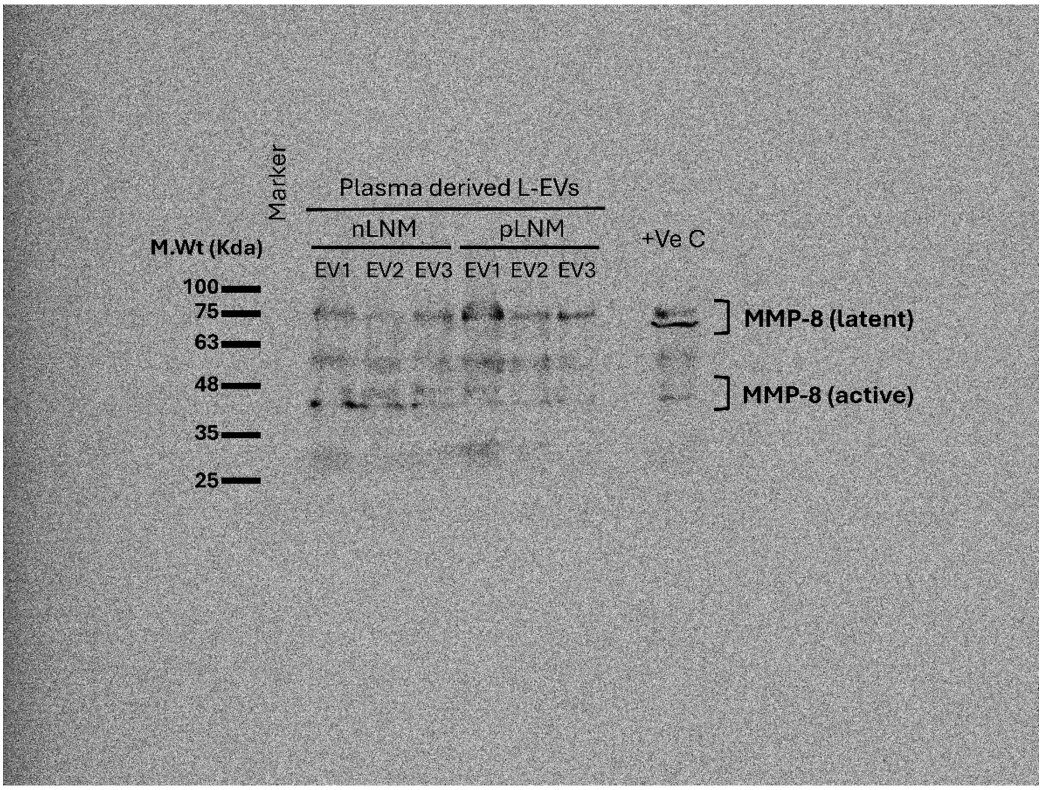

Figure S19. Raw data for Fig. 3a. Second original western blot image of L-EV-derived MMP-8 using different independently prepared pooled L-EV samples from each group (nLNM: EV1 (n = 4), EV2 (n = 4) and EV3 (n = 5); pLNM: EV1 (n = 3), EV2 (n = 4) and EV3 (n = 4)).

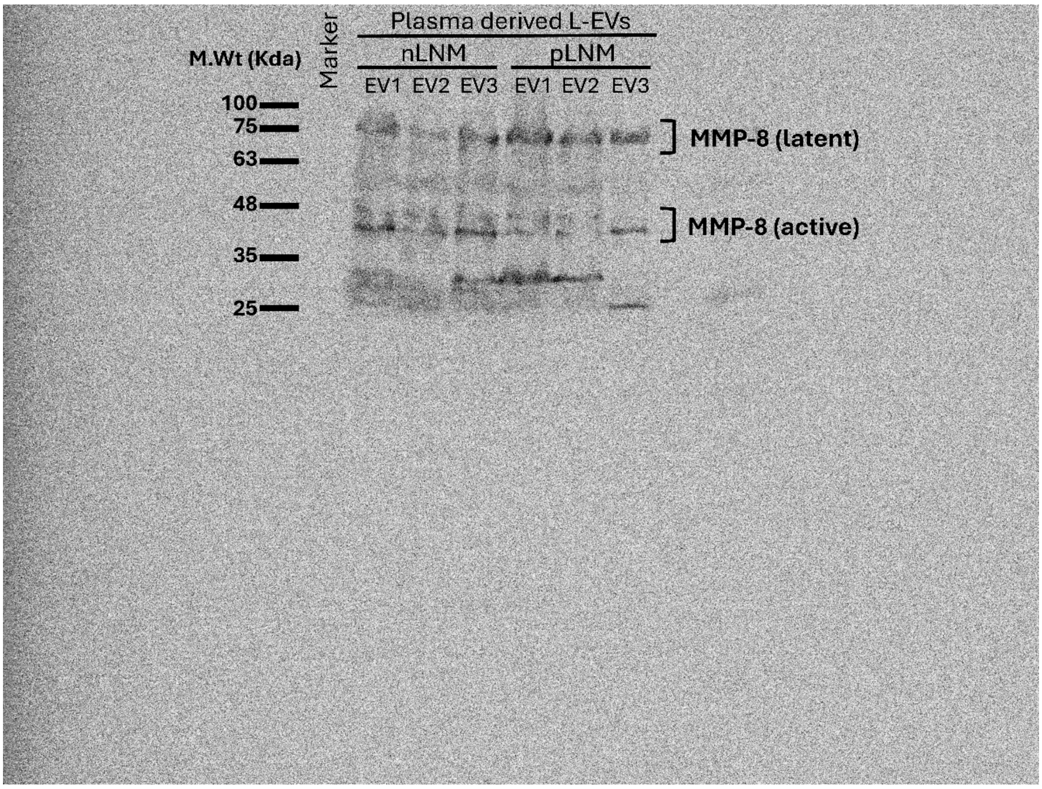

Figure S20. Raw data for Fig. 3a. Third original western blot image of L-EV-derived MMP-8 using different independently prepared pooled L-EV samples from each group (nLNM: EV1 (n = 4), EV2 (n = 4) and EV3 (n = 5); pLNM: EV1 (n = 3), EV2 (n = 4) and EV3 (n = 4)).

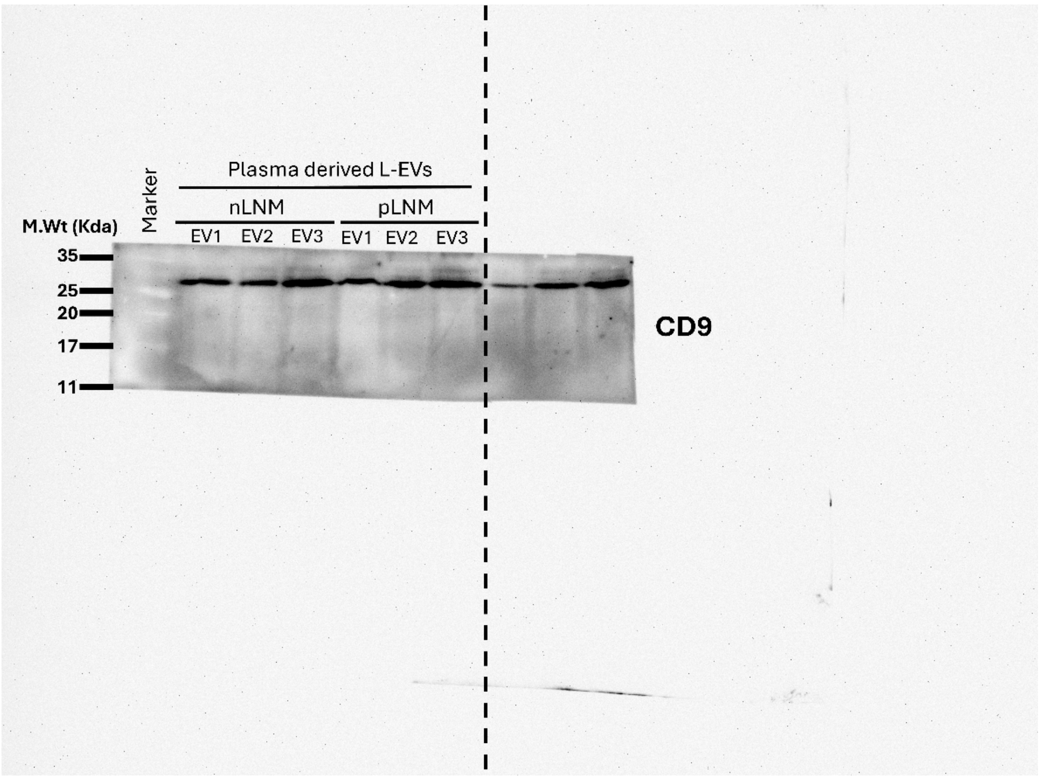

Figure S21. Raw data for Fig. 3a. Original western blot image of L-EV-derived CD9 using independently prepared pooled L-EV samples from each group (nLNM: EV1 (n = 4), EV2 (n = 5) and EV3 (n = 5); pLNM: EV1 (n = 3), EV2 (n = 3) and EV3 (n = 4)).

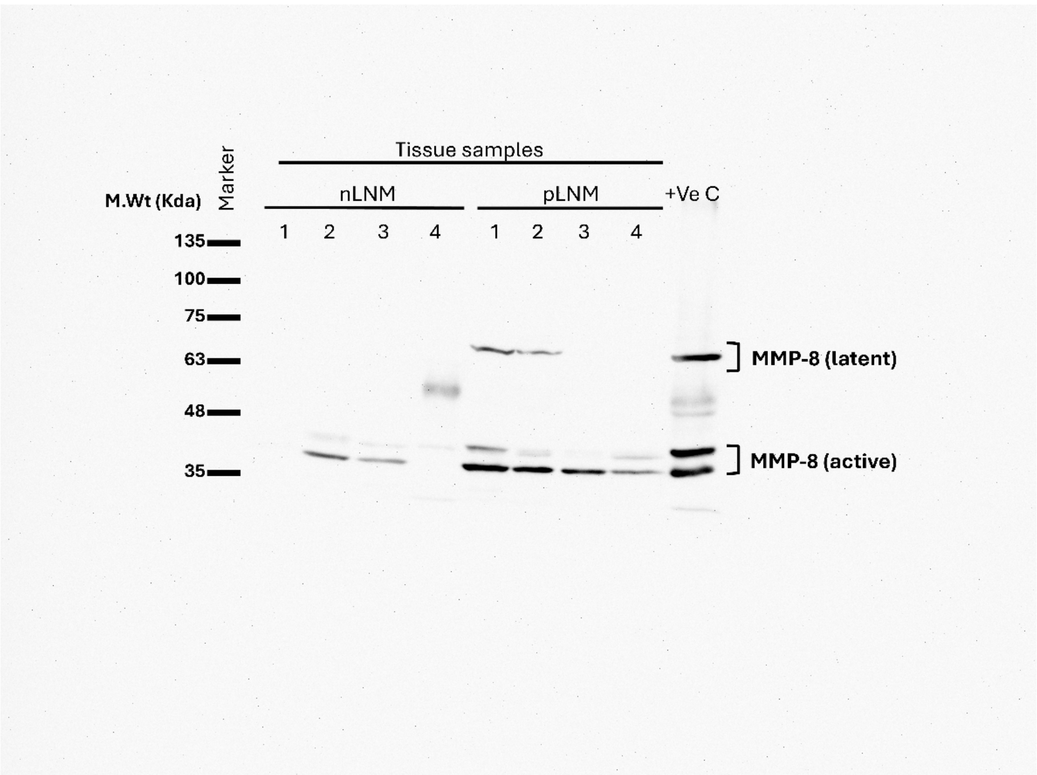

Figure S22. Raw data for Fig. 3c. First original western blot image of tumor-derived MMP-8 in nLNM (n = 4) and pLNM (n = 4) patients.

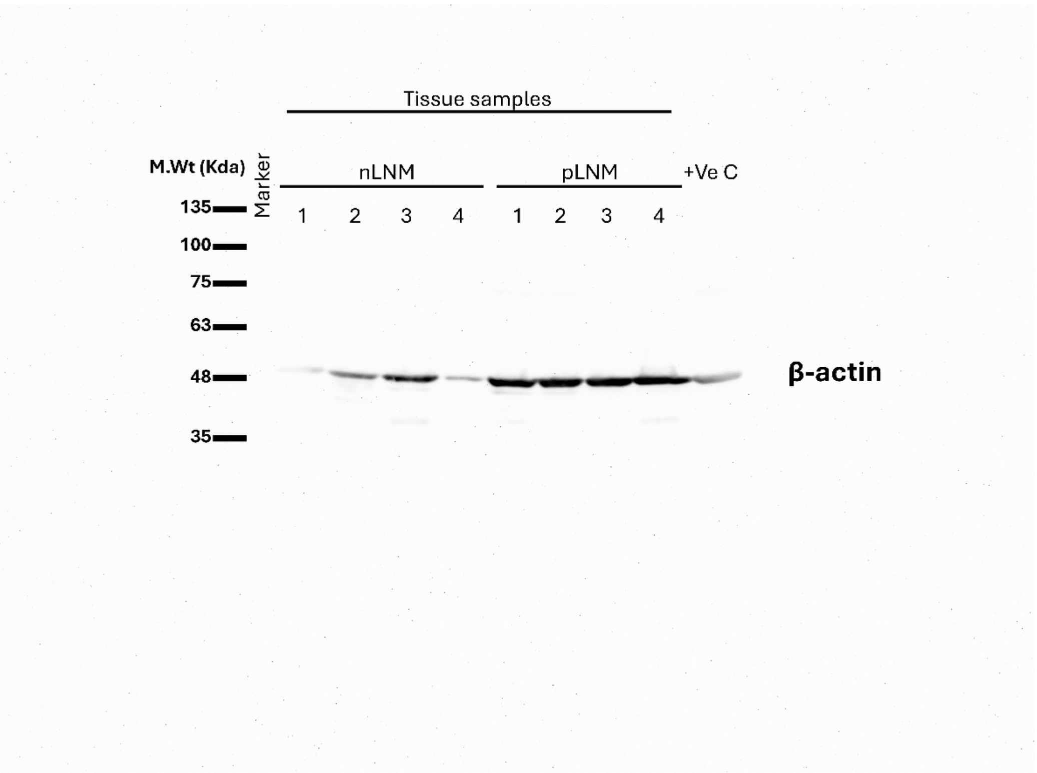

Figure S23. Raw data for Fig. 3c. First original western blot image of tumor-derived β-actin in nLNM (n = 4) and pLNM (n = 4) patients.

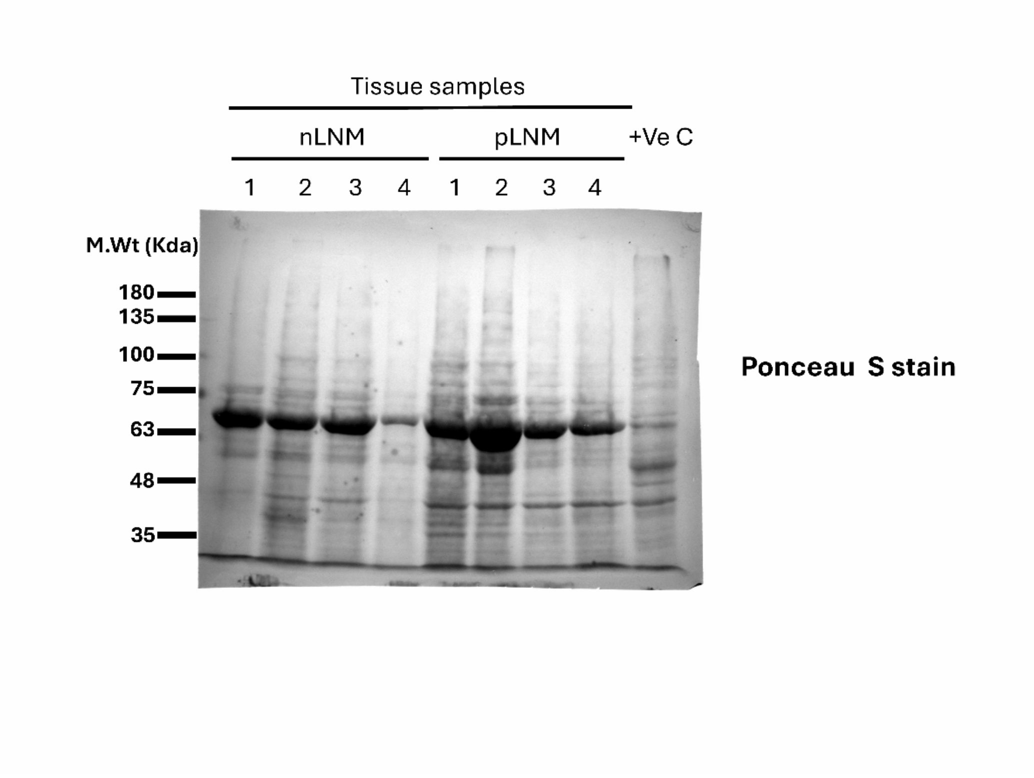

Figure S24. Raw data for Fig. 3c. First original image of Ponceau S stain of the corresponding membrane as a total protein loading in nLNM (n = 4) and pLNM (n = 4) patients.

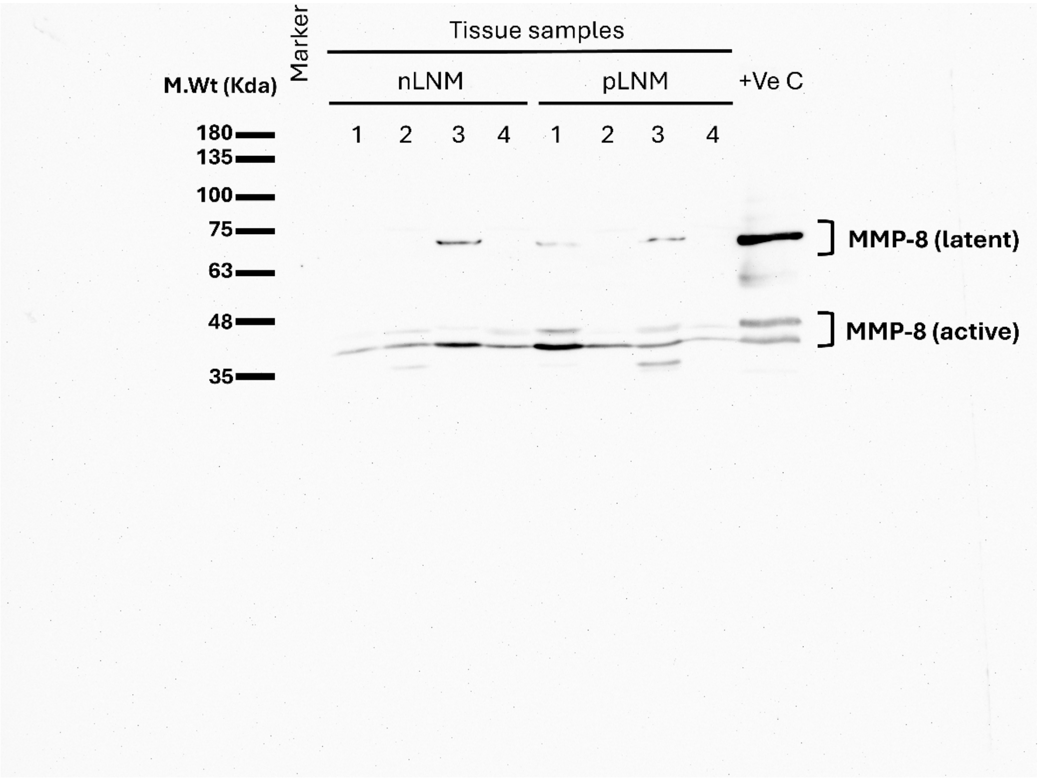

Figure S25. Raw data for Fig. 3c. Second original western blot image of tumor-derived MMP-8 in different nLNM (n = 4) and pLNM (n = 4) patients.

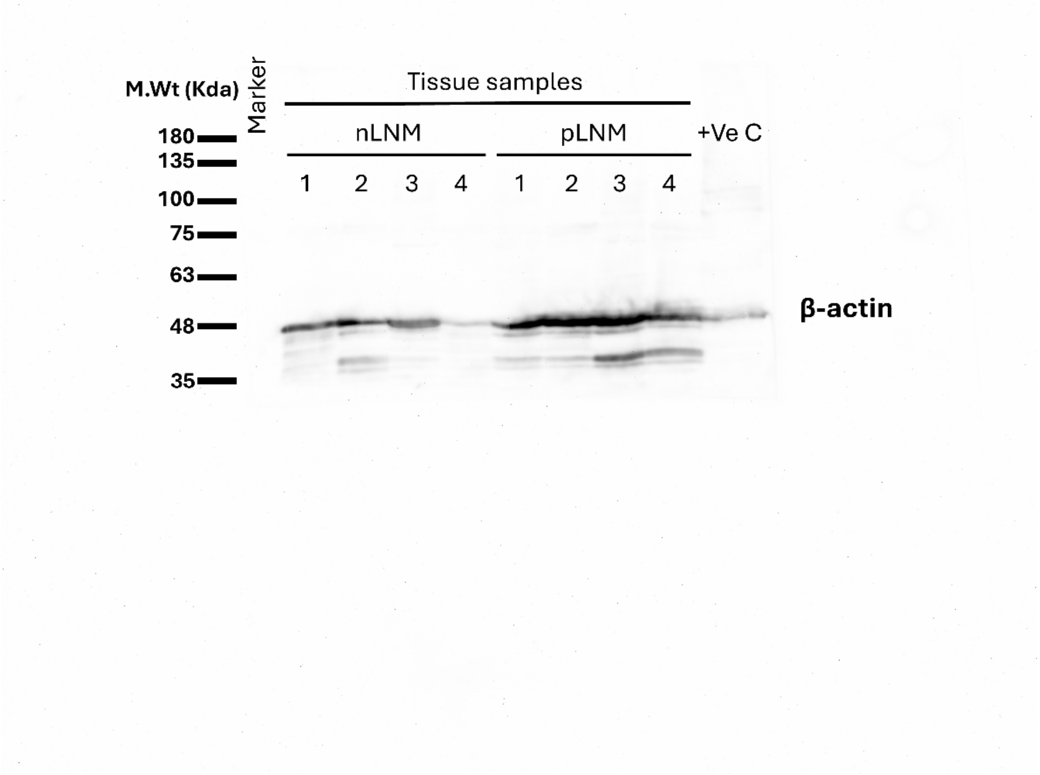

Figure S26. Raw data for Fig. 3c. Second original western blot image of tumor-derived β-actin in different nLNM (n = 4) and pLNM (n = 4) patients.

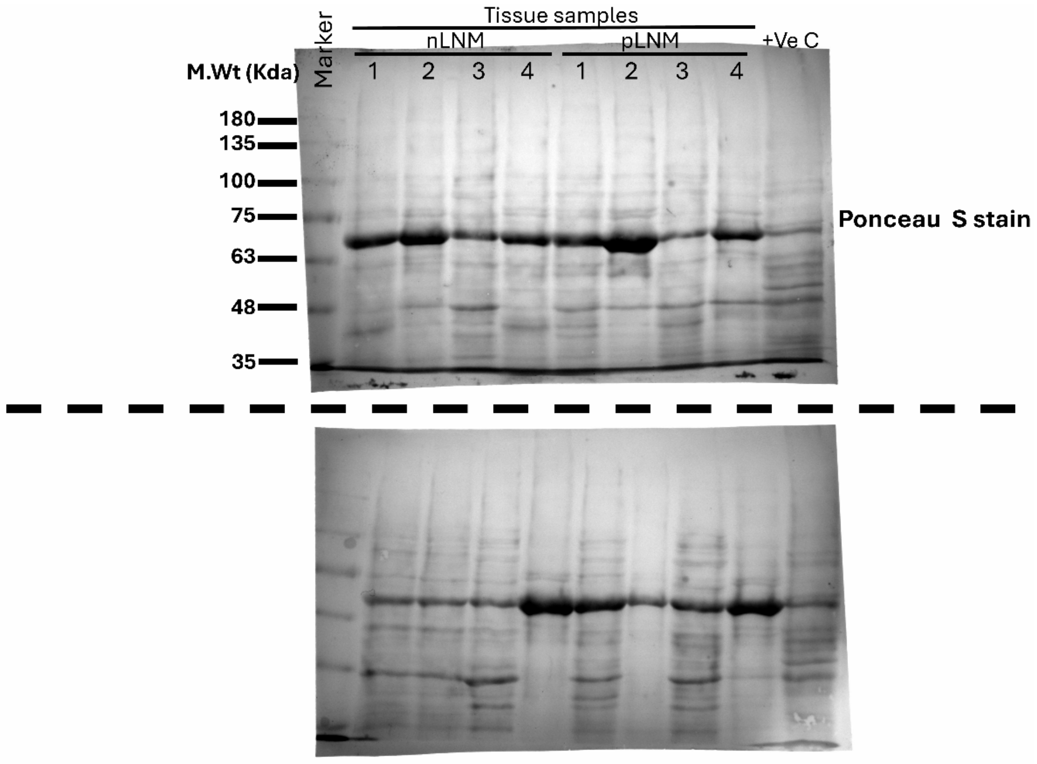

Figure S27. Raw data for Fig. 3c. Second original image of Ponceau S stain of the corresponding membrane as a total protein loading in different nLNM (n = 4) and pLNM (n = 4) patients.

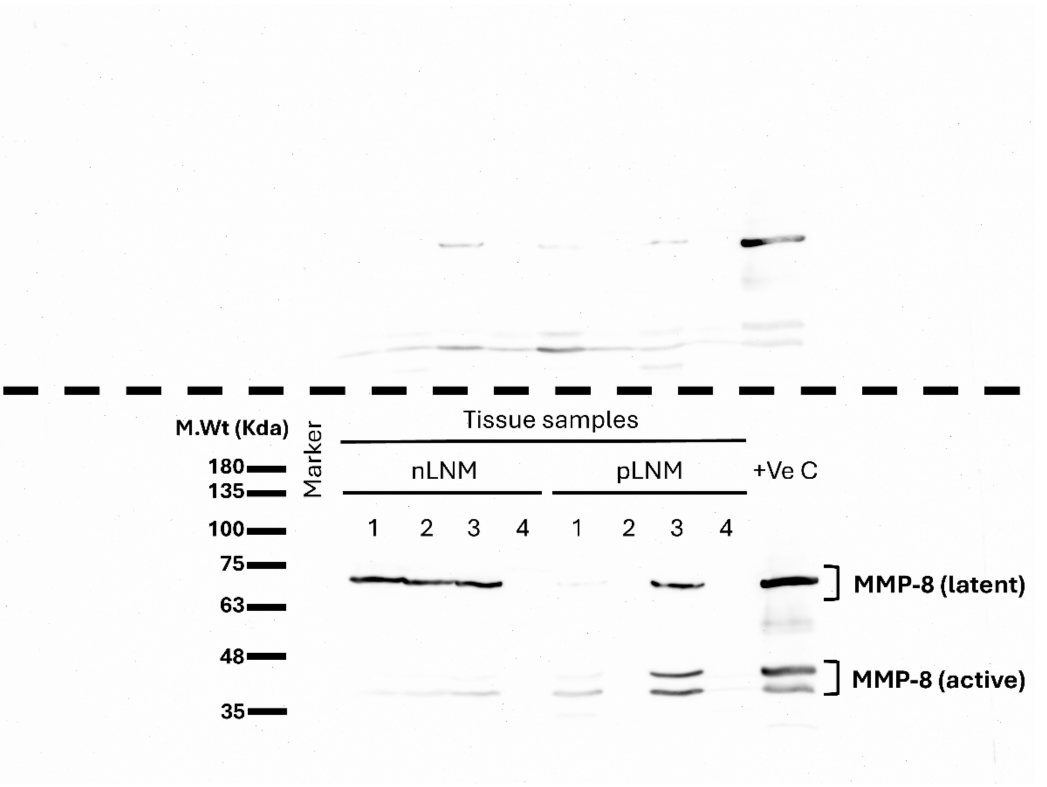

Figure S28. Raw data for Fig. 3c. Third original western blot image of tumor-derived MMP-8 in different nLNM (n = 4) and pLNM (n = 4) patients.

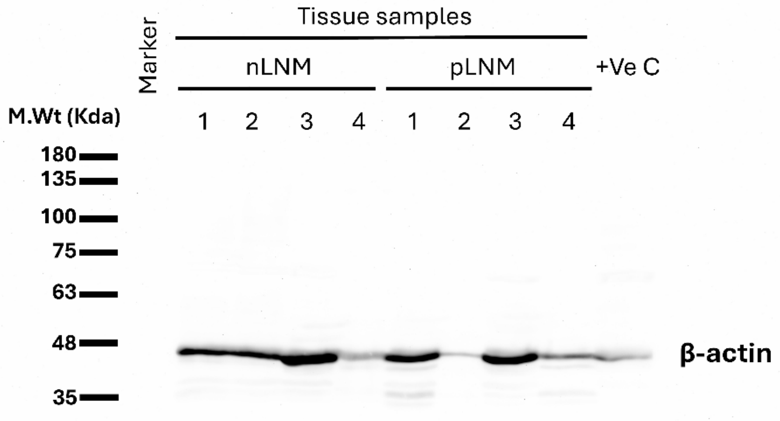

Figure S29. Raw data for Fig. 3c. Third original western blot image of tumor-derived β-actin in different nLNM (n = 4) and pLNM (n = 4) patients.

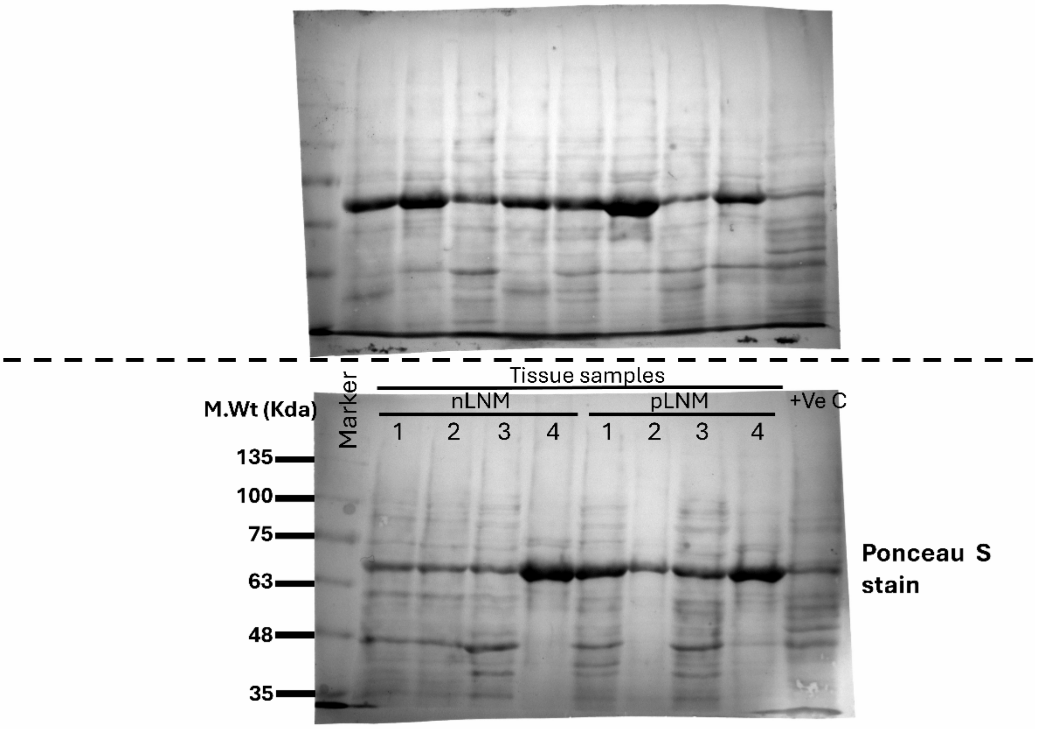

Figure S30. Raw data for Fig. 3c. Third original image of Ponceau S stain of the corresponding membrane as a total protein loading in different nLNM (n = 4) and pLNM (n = 4) patients.

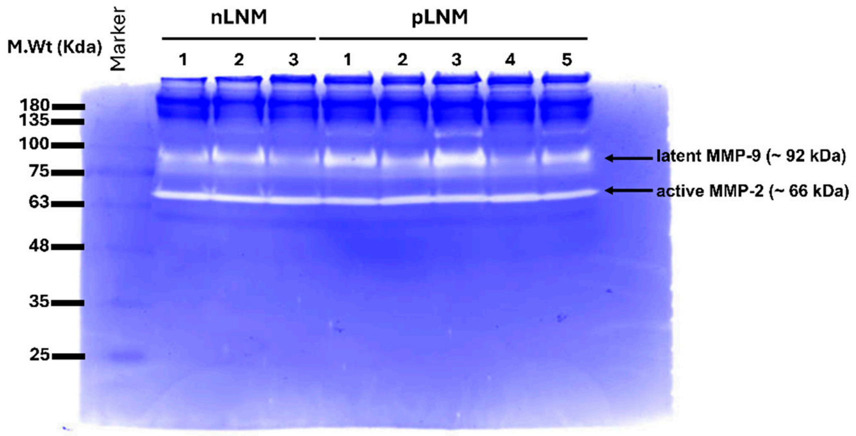

Figure S31. Raw data for Fig. 4a. Original zymography image of plasma samples derived from nLNM (n = 3) and pLNM (n = 5) patients.

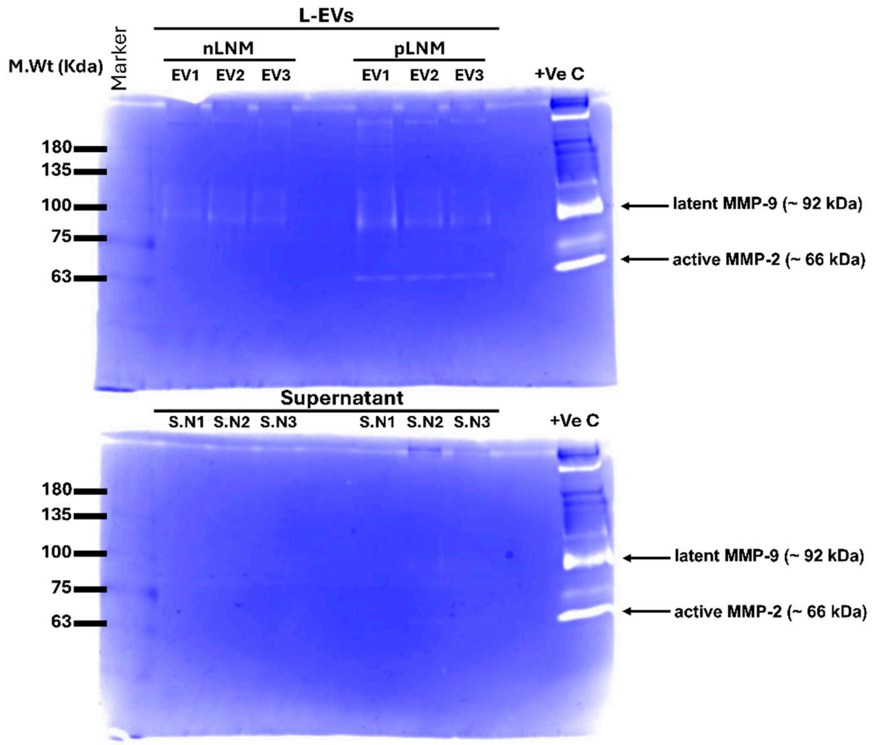

Figure S32. Raw data for Fig. 4c&e. First original zymography image of L-EV samples using independently prepared pooled L-EV samples from each group (nLNM: EV1 (n = 6), EV2 (n = 7) and EV3 (n = 7); pLNM: EV1 (n = 5), EV2 (n = 5) and EV3 (n = 6)) and the corresponding post-pelleting supernatant fraction samples.

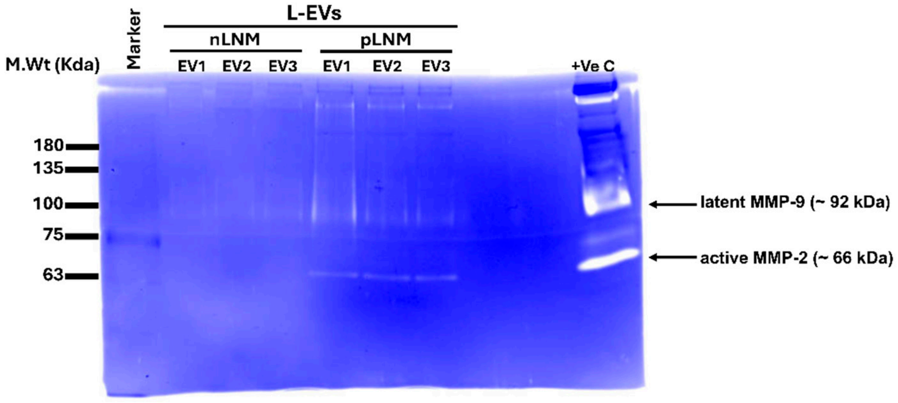

Figure S33. Raw data for Fig. 4c. Second original zymography image of L-EV samples using different independently prepared pooled L-EV samples from each group (nLNM: EV1 (n = 6), EV2 (n = 7) and EV3 (n = 7); pLNM: EV1 (n = 5), EV2 (n = 5) and EV3 (n = 6)).

Table S1. List of antibodies used in this study.

Antibody	Working dilution	Manufacturer	City and Country
CD9	1:1000	Invitrogen (Ts9)	Carlsbad, USA
HSP70/HSC70	1:1000	Santa-Cruz (W27): sc-24	Dallas, TX, USA
ALIX	1:1000	Santa-Cruz (1A12): sc-53540	Dallas, TX, USA
Calnexin	1:1000	Santa-Cruz (H-70): sc-11397	Dallas, TX, USA
MMP-8	1:1000	Santa-Cruz (B-1): sc-514803	Dallas, TX, USA
β-Actin	1:1000	Santa-Cruz (C4): sc-47778	Dallas, TX, USA
Goat anti-mouse	1:2000	KPL (Cat: 04-18-06)	Gaithersburg, MD, USA
Goat Anti-Rabbit IgG, H & L chain specific peroxidase conjugate	1:2000	Merckmillipore (Cat: 401353)	Darmstadt, Germany

Table S2. List of primers used in this study.

Gene		Sequence
MMP-2	F	GCCGTGTTTGCCATCTGTTT
	R	CTGCAGGGAGCAGAGATTCG
MMP-8	F	CAACCTACTGGACCAAGCACAC
	R	TGTAGCTGAGGATGCCTTCTCC
18S	F	AACCCGTTGAACCCCATT
	R	CCATCCAATCGGTAGCG

Disclaimer/Publisher's Note: The statements, opinions and data contained in all publications are solely those of the individual author(s) and contributor(s) and not of MDPI and/or the editor(s). MDPI and/or the editor(s) disclaim responsibility for any injury to people or property resulting from any ideas, methods, instructions or products referred to in the content.

https://doi.org/10.3390/cancers18091461
